# Loss of PTB or Negative Regulation of Notch mRNA Reveals Distinct Zones of Notch and Actin Protein Accumulation in Drosophila Embryo

**DOI:** 10.1371/journal.pone.0021876

**Published:** 2011-07-05

**Authors:** Cedric S. Wesley, Heng Guo, Kanita A. Chaudhry, Markus J. Thali, Jerry C. Yin, Todd Clason, Umadevi V. Wesley

**Affiliations:** 1 Departments of Genetics and Medical Genetics, University of Wisconsin, Madison, Wisconsin, United States of America; 2 Department of Biochemistry, Boston University, Boston, Massachusetts, United States of America; 3 Department of Biochemistry, University of Vermont, Burlington, Vermont, United States of America; 4 Department of Microbiology and Molecular Genetics, University of Vermont, Burlington, Vermont, United States of America; 5 Department of Anatomy and Neurobiology, University of Vermont, Burlington, Vermont, United States of America; 6 Department of Neurological Surgery, University of Wisconsin, Madison, Wisconsin, United States of America; Fred Hutchinson Cancer Research Center, United States of America

## Abstract

Polypyrimidine Tract Binding (PTB) protein is a regulator of mRNA processing and translation. Genetic screens and studies of wing and bristle development during the post-embryonic stages of Drosophila suggest that it is a negative regulator of the Notch pathway. How PTB regulates the Notch pathway is unknown. Our studies of Drosophila embryogenesis indicate that (1) the *Notch* mRNA is a potential target of PTB, (2) PTB and Notch functions in the dorso-lateral regions of the Drosophila embryo are linked to actin regulation but not their functions in the ventral region, and (3) the actin-related Notch activity in the dorso-lateral regions might require a Notch activity at or near the cell surface that is different from the nuclear Notch activity involved in cell fate specification in the ventral region. These data raise the possibility that the Drosophila embryo is divided into zones of different PTB and Notch activities based on whether or not they are linked to actin regulation. They also provide clues to the almost forgotten role of Notch in cell adhesion and reveal a role for the Notch pathway in cell fusions.

## Introduction

Polypyrimidine Tract Binding (PTB) protein (also known as Heterogeneous Nuclear RibonucleoProtein I or hnRNP I) plays critical roles in mRNA metabolism. It contains four RNA Recognition Motifs (RRMs) and binds to pyrimidine-rich sequences with motifs such as UCUUC and CUCUCU. Biochemical studies, most of them performed using mammalian *in vitro* and cultured cell systems, indicate that PTB regulates (often negatively) mRNA splicing, 3′ processing, stabilization, nuclear export, subcellular localization, and translation [1]–[11]. PTB in *Drosophila melanogaster* (*dmPTB*) is encoded by the *hephaestus* locus. Genetic screens and developmental studies performed on post-embryonic stages of this organism indicate that PTB regulates oogenesis in adult females [12], spermatogenesis in adult males [13], and sensory bristle and wing margin development during larval and pupal stages [14]–[17]. Studies of wing development, which include a large-scale genetic screen and an analysis of somatic clones of mutant cells, indicate that *hephaestus* is a negative regulator of Notch pathway activities [15], [17]. In *hephaestus* somatic mutant clones the level of active Notch molecule (the Notch intracellular domain, see below) is known to increase [17] but it is not known whether that is a direct or an indirect effect. In other words, neither the Notch pathway target nor the underlying mechanism is known. Our recent studies show that the *Notch* gene is negatively regulated at the level of mRNA 3′ processing during Drosophila embryogenesis [18], [19] raising the possibility that PTB regulates *Notch* mRNA and protein production during development.

The Notch protein is a cell surface receptor that is required for the development of all animal tissues. At any particular developmental stage, it is involved in the differentiation of many different tissues in different regions. The mechanism underlying Notch function is generally thought to be the same everywhere: In response to binding of a ligand such as Delta, the Notch receptor is proteolytically cleaved to release the Notch intracellular domain (N^intra^/NICD) from the cell surface. N^intra^/NICD translocates to the nucleus, associates with transcription factors including Suppressor of Hairless and Mastermind, and turns on transcription of target genes. A Mastermind-dependent process degrades N^intra^/NICD, possibly to keep transcription commensurate with the amount of N^intra^/NICD production. This mechanism, called canonical Notch signaling, is used as a binary switch during cell fate specification. Cells that block N^intra^/NICD production commit to one fate (generally the default fate) and cells that produce N^intra^/NICD acquire the alternative cell fate. Depending on the developmental event or context, N^intra^/NICD [20]–[30] targets different sets of effector genes.

There is suggestive evidence in assorted systems that some Notch functions are not based on canonical Notch signaling. Many of these functions, particularly those related to actin, cytoskeletion, or extracellular matrix appear to be based on a mechanism that does not involve Suppressor of Hairless or N^intra^/NICD that are required for canonical Notch signaling [31]–[38]. The mechanism(s) underlying these non-canonical Notch functions is(are) poorly understood (e.g. [33]). As a consequence, we do not know much about the molecular features associated with these functions. For example, we do not know whether N^intra^/NICD production is a part of the known non-canonical Notch functions. We also do not know whether the canonical and the non-canonical Notch activities function everywhere in the developing animal (together or in tandem) or are confined to distinct regions of the developing animal.

The ventral region of the Drosophila embryo was the region where the role of Notch in cell fate specification was first discovered about 70 years ago [39]. Ever since then it has served as an excellent model for the study of the function and mechanism of canonical Notch signaling. Features first identified here were subsequently found in all animals. The development of the Central Nervous System (CNS) is initiated in the ventral region, within clusters of proneural cells that have acquired the potential to become neuronal cells. Most proneural cells activate canonical Notch signaling that promotes the expression of Enhancer of split Complex (E(spl)C) genes and become the epidermal precursor cells. The remaining cells block this signaling and the expression of E(spl)C genes and become the neuronal precursor cells. The epidermal precursor cells remain in the periphery of the embryo and differentiate the epidermis that includes a series of actin-rich denticle belts. The neuronal precursor cells migrate interiorly and differentiate into the CNS with elaborate actin-based processes (e.g., axons).

The dorso-lateral regions of the developing Drosophila embryo support many processes that require the cell fate specification functions of Notch, for example the specification of neuronal precursor cells that differentiate the peripheral nervous system or the pericardial cells that differentiate the dorsal vessel (heart). These regions are also involved in actin-based processes such as gastrulation and dorsal closure processes. There are no published reports of the role of Notch in these actin-based processes. It is possibly because of epistasis: loss of Notch function suppresses the production of epidermal cells that are required for many aspects of gastrulation and dorsal closure [40], [41]. In any case, both the ventral and the dorso-lateral regions of the Drosophila embryo are involved in cell fate specification and actin-based processes. Consequently, both the regions were expected to have the same potential for canonical and non-canonical Notch signaling and manifest similar molecular features associated with Notch protein activities. If *hephaestus* negatively regulated *Notch* functions in embryos (as it does during wing development), its loss of function was expected to have a similar effect on Notch features in the ventral and the dorso-lateral regions.

Results reported here show that in mutant *hephaestus* embryos the level of Notch mRNA and protein activity is increased indicating that Notch mRNA might be targeted by the Hephaestus protein for negative regulation. Remarkably, in *hephaestus* embryos the Notch protein accumulates at or near the cell surface in the dorso-lateral regions but not in the ventral region. Notch accumulation in the dorso-lateral regions is associated with actin accumulation, cell fusions, and disruption in dorsal closure and cardiogenesis processes. The same phenomenon is observed in mutant *Notch* embryos that have lost negative regulation at the level of mRNA 3′ processing suggesting that the *hephaestus* phenotypes are possibly consequences of increased Notch activities. Over-expression of N^intra^, thereby canonical Notch signaling, recapitulates the molecular and morphological phenotypes in the ventral regions of mutant *hephaestus* or *Notch* embryos but not the phenotypes in the dorso-lateral regions. These data suggest that the Drosophila embryo is zoned based on whether or not PTB and Notch activities are directly linked to actin-based processes. Thus, cells that appear to be the same (epidermal cells) and part of the same epithelial layer might differ in developmental potential depending on their place of origin in the embryo (ventral or the dorso-lateral regions).

## Materials and Methods

*hephaestus* alleles used in our studies are *heph^03429^* and *heph^2^*. They are loss of function alleles due to P element insertions. *heph^03429^* appears to be the stronger allele as its homozygotes do not survive to adulthood but a few *heph^2^* homozygotes do ([13], [17], FlyBase). *heph* stocks were obtained from Bloomington Stock Center. They contain a TM3 Sb^1^ balancer and not a TM3 Sb^1^ Ser^1^ balancer as stated in the description sheet. Our studies also indicate that the expressivity of zygotic phenotypes of *heph^03429^* is drastically reduced if the mother is heterozygous for *Notch* or *Serrate* null alleles. For example, in the background of the TM3actGFPSer^1^ balancer the accumulation of actin in the dorso-lateral regions of *heph^03429^* embryos that manifests in embryonic stage 14 is delayed until embryonic stage 17 (**Fig. S1**). The *yellow white* (*yw*) fly stock served as the wild type (WT) control. The Notch null mutants used are *N^55e11^* or *N^264-47^*; the Notch gain-of-function allele used was Nnd1-dse [18], [19]. Zygotic *heph^03429^*; *N* ^-^ embryos were obtained using standard genetic crosses and stocks with green (GFP) and blue (lacZ) balancers. Stocks were maintained at 18°C and experimental embryos were collected at room temperature (23°C to 27 °C) or 29–30°C (*yw* control and N^nd1-dse^) over a 0–24-hour period. They were processed immediately for immuno-labeling studies or aged for an additional day or two before processing (periods for stock raised at 18°C or 29–30°C were corrected for the difference in developmental rate). Embryos were immuno-labeled with antibodies against Notch [25], Hunchback (gift from Paul MacDonald), Kruppel (gift from John Reinitz), Pericardin (EC11, DSHB), or Actin (Abcam, ab49846), following standard protocols [42], [43]. The nuclear stain DAPI was included where required. Embryos were devitellinized by hand for phalloidin labeling. Acridine orange hydrochloride hydrate (Acros Organics, 423340010) was used to detect apoptosis in embryos using the protocol described in [44]. Embryos were sorted using green/blue balancers and/or morphological phenotypes.

RNA expression was analyzed by northern blotting [24], [27]. For exogenous expression of N^intra^/NICD, we used a UAS-N^intra^/NICD transgene [45] and *daughterless* Gal4 (daGal4) or *heat shock protein 70* Gal4 (hsGal4) drivers provided through the males. hsGal4 flies and embryos were reared at 18°C and transferred to 30°C at different stages. All offspring embryos would express N^intra^/NICD, as homozygous parents were used.

Although similar results were obtained with imuno-fluorescence, detailed studies were done with immuno-cytochemical procedures (based on Horse Radish Peroxidase or Alkaline Phosphatase activity) because they were efficient and cost-effective, enabling us to examine simultaneously thousands of embryos and determine stage-specific expressivity and penetrance of mutant phenotypes. Although Phalloidin and actin antibody gave similar results, we relied on the latter for double labeling studies because it was less tedious. Wild type and mutant embryos were processed identically and mounted in Phosphate Buffered Saline with 0.5% triton×100 (PBT) using strips of glass cover-slip as props so that embryos could be rolled to desired position for imaging.

For determining the effect of Notch and Delta interaction on F-actin in cultured cells, cell aggregations were performed using S2 cells expressing heat shock inducible Notch or Delta and procedures described in [26]–[28]. Cells were fixed with paraformaldehyde and processed for labeling using antibodies against Notch and Delta, and Phalloidin as the probe for F-actin (AlexaFluor 568-Phalloidin). *In vitro* cell fusion assays were performed with clone-8 cells that endogenously express Notch (but not Delta), Schneider (S2) cells that express neither Notch nor Delta [23], [24], and S2 cells that constitutively express Delta under the control of the Drosophila *actin5C* promoter (S2-actDelta). For generating the S2-actDelta cell line, *actin5C* promoter and the *Delta* cDNA were cloned the into pUAST vector (please see [18], [19]) and a stable S2 line established by co-transfecting with it with pCopHygro plasmid [43]. Near confluent cells were washed and resuspended at a concentration of 3–5×10^6^ cells per ml in amine-free buffer (IP buffer in [23], [24]) with CMTPX (CellTracker Red) or CMFDA (CellTracker Green) (see Molecular Probes manual MP 02925 for details of the dyes). Clone-8 cells were treated with 1 µM CMTPX (CellTracker Red) and S2-actDelta and S2 cells were treated with 10 µM CMFDA (CellTracker Green), incubated in the dark for 45 minutes, washed once with 1 ml IP buffer, resuspended in 2 ml of cell culture medium (with FBS), and incubated in the dark for 2 hours to complete dye activation and removal of un-reacted dye molecules. The cells were pelleted and re-suspended in cell culture medium (with FBS) at a concentration of 3×10^6^/ml. Varying cell concentration controlled the size of cell aggregates. 200 µl of Red-clone 8 cells were mixed with 200 µl of Green-S2-actDelta cells or Green-S2-Cells in a microfuge tube and immediately transferred to a 12-well tissue culture-treated microplate. The plates were gently rotated for 5–10 minutes until Notch and Delta driven cell aggregates became apparent. The plates were then covered in aluminum foil and incubated without shaking in the dark for various time periods (2 hours to 2 days).

DeltaVision imaging was done at the Neuroscience Core Imaging facility, University of Vermont College of Medicine. All other imaging was done using an upright Nikon SMZ1500 stereoscope or a Nikon 2500 inverted fluorescence microscope fitted with a Spot RT Slider CCD camera. Wild type and mutant embryos were imaged together or with identical settings. Extra Long Working Distance (ELWD) 40×objective was used to image live cells through the bottom of the microplate (total magnification was 400X). Images were processed using Photoshop (Adobe) and Canvas (Deneba) programs. Any adjustment made to brightness/contrast was applied to the whole image and the same settings were used for all compared samples.

## Results

### 1. Neurogenesis is suppressed in *hephaestus* null embryos

**Figure 1:**
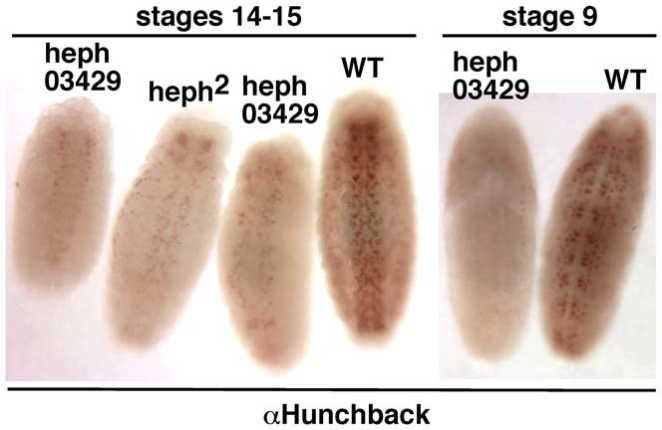
Mutant *heph* embryos manifest suppression of the CNS development in the ventral region, a phenotype linked to excess canonical Notch signaling phenotype. Expression of Hunchback, a well-known neuronal marker, was used to assess the CNS development. Hunchback expression in Notch null and gain-of-canonical Notch signaling (N^intra^/NICD) embryos is shown in Figure S2. Suppressed neurogenesis phenotype became apparent as early as stage 9 (about four hours of embryogenesis) and was severe at stage 14–15 (about 14–16 hours of embryogenesis). Variation in the suppression of neurogenesis in *heph* embryos was observed, which is presumably due to variable maternal contribution. All embryos shown were from the same experiment and were processed identically.

**Figure 2:**
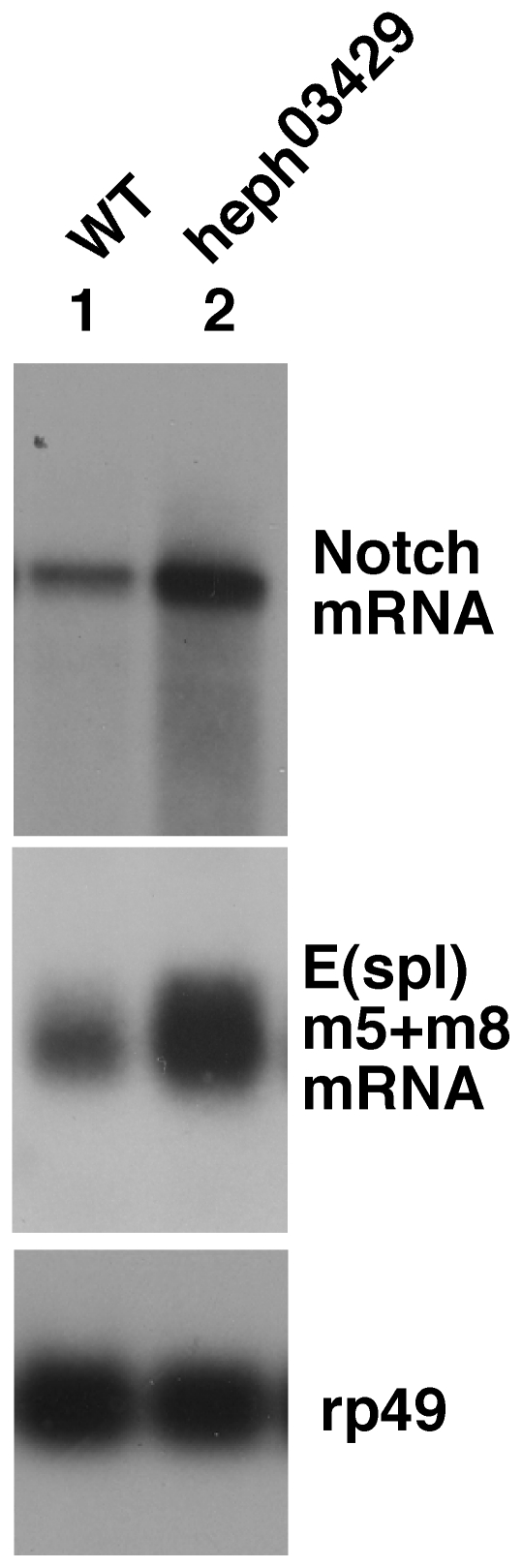
RNA of *Notch* and *E(spl)* genes *m5* and *m8*, the latter targets of canonical Notch signaling, were over- expressed in *heph^03429^* embryos. RNAs used on northern blots were extracted from 3 to 6 hours old embryos that manifest peak canonical Notch signaling activity related to the CNS development. rp49 is the loading control.

To determine if the loss of *hephaestus* function affected *Notch* function during embryogenesis, we examined the CNS development in mutant *hephaestus* embryos as this process is under the control of canonical Notch signaling and is the most widely accepted assay for this signaling. The neuronal precursor cells and many of their progeny express the neuronal marker protein Hunchback. When there is loss of canonical Notch signaling, as in Notch null embryos, an excess of Hunchback signal is observed. When there is an excess of this signaling, as in transgenic embryos over-expressing N^intra^/NICD from an exogenous promoter, a suppression of Hunchback signal is observed (**Fig. S2**). Examination of *heph^03429^* and *heph^2^* embryos showed that Hunchback expression is suppressed (**Fig. 1**). Suppression of neurogenesis was apparent as early as stage 9 and became progressively severe with age. As expected from the difference in the strengths of the alleles, the phenotype in *heph^03429^* embryos was much stronger than in *heph^2^* embryos. While almost all *heph^03429^* embryos showed defective CNS developments, only about 20% of *heph^2^* embryos showed defective CNS. Thus, two independently isolated alleles manifested similar phenotypes indicating that these phenotypes are linked to the *hephaestus* gene. We next examined if *Notch* mRNA expression is affected in *heph* mutant embryos. For this purpose we used total RNA extracted from 3 to 6 hour-old *heph^03429^* embryos which would be most active for canonical Notch signaling related to neurogenesis (specification of the epidermal precursor cells). Results showed that *heph^03429^* embryos express a high level of Notch mRNA as well as high levels of E(spl)C mRNA, the target of canonical Notch signaling during neurogenesis (**Fig. 2**). Based on these studies we conclude that the loss of *hephaestus* function results in increased Notch mRNA expression and possibly as a consequence increased Notch activity that suppresses the CNS development. This conclusion is consistent with studies of wing development showing that *hephaestus* is a negative regulator of Notch activity [14], [15], [17]. The increased level of *Notch* mRNA in *heph^03429^* embryos suggests that the *Notch* gene is a target of *hephaestus* function related to negative regulation of the Notch pathway.

### 2. Mutant *hephaestus* embryos are defective in dorsal closure

**Figure 3:**
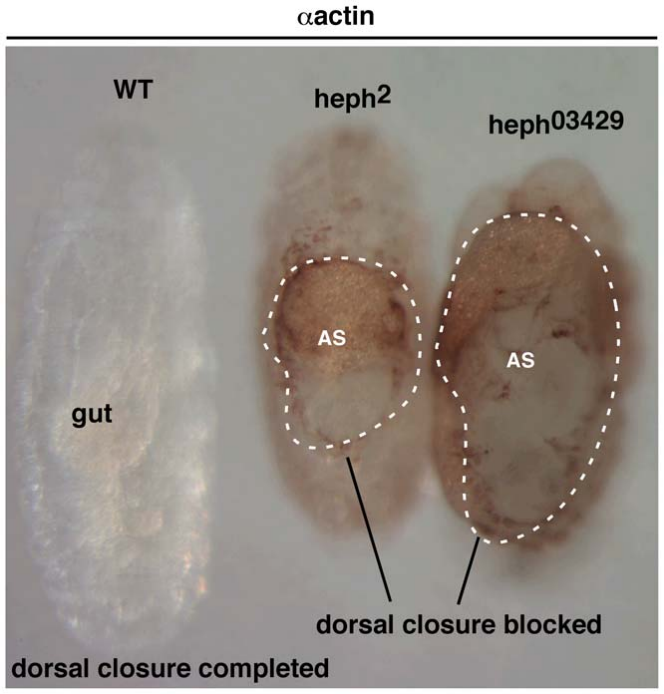
Dorsal closure process is blocked in mutant *heph* embryos. While in the wild type embryo the dorsal region was closed and the extra-embryonic amnioserosa (AS) was completely eliminated by about 22 hours after egg laying (left), in *heph^03429^* and *heph^2^* embryos the dorsal closure process was incomplete and AS persisted even after 48 hours. Embryos were labeled using the actin antibody. All embryos/larva shown were from the same experiment and were processed identically.

Our studies also revealed that the dorsal closure process is severely disrupted in *hephaestus* mutant embryos. For much of wild type embryogenesis the extra-embryonic aminoserosa occupies the dorsal region of the embryo. Aminoserosal cells help the dorso-lateral epidermal cells move to the dorsal midline for fusion and closure of the embryo. They also undergo apoptosis in a progressive manner to accommodate the advancing lateral epidermis [40]. In *heph^03429^* embryos the dorsal closure process was blocked and the cuticle poorly developed even a day after embryogenesis had ended in the wild type embryos (**Fig. 3**). The wild type embryo shown has just completed embryogenesis (∼22 hours, end of stage 17). Its dorsal closure process is complete, internal organs are conspicuous (e.g. the gut), and cuticle is fully developed into the rubbery exoskeleton that resists immuno-staining (as antibodies cannot penetrate the tough cuticle). The mutant *heph^03429^* and *heph^2^* embryos shown are more than a day older (∼48 hours). In these embryos the amnioserosa (AS) persists in the dorsal region and most of other embryogenesis events are arrested. Although the amnioserosa in *heph* mutant embryos retains the morphology (and can be easily identified using it), its physiology appeared to be altered as expression of markers such as Kruppel [46] was drastically reduced. While most *heph^03429^* embryos appeared to cease development at about stage 14 to 16 (12–16 hours of embryogenesis), only about 20% of *heph^2^* embryos appeared to cease development at these stages. These determinations are gross approximations as not all parts of the dead embryos ceased development at the same stage (i.e. the embryos were essentially developmental mosaics). Embryos that had ceased development died of necrosis between 48–72 hours after egg laying.

### 3. Mutant *hephaestus* embryos are defective in cardiogenesis

To determine whether the defect in the dorso-lateral lateral region of *heph* mutant embryos is specific to the dorsal closure process, we examined development of the dorsal vessel. Its development is initiated shortly after gastrulation and well before the initiation of the dorsal closure process. Pairs of cardioblasts develop on either side of the embryo, from the lateral myoblast cells along the anterior-posterior axis. Notch activity in one cell of each pair suppresses the cardioblast fate and specifies the alternate pericardial cell fate. While cardioblasts are responsible for producing the dorsal vessel proper, pericardial cells are responsible for producing support structures and for secreting the extracellular matrix protein Pericardin that is required for attaching the cardioblasts to the dorso-lateral epidermal cells. The two longitudinal rows of pericardial cell-cardioblast pairs that form on either side of the embryo migrate to the dorsal midline by hitchhiking on the dorso-lateral epidermal cells involved in dorsal closure. After reaching the dorsal midline, they fuse and differentiate the dorsal vessel. Notch activity is also required for this differentiation [47]–[51]. In embryos lacking Notch function (*Notch* null embryos), Pericardin is not expressed, as pericardial cells are not formed (**Fig S3**).

**Figure 4:**
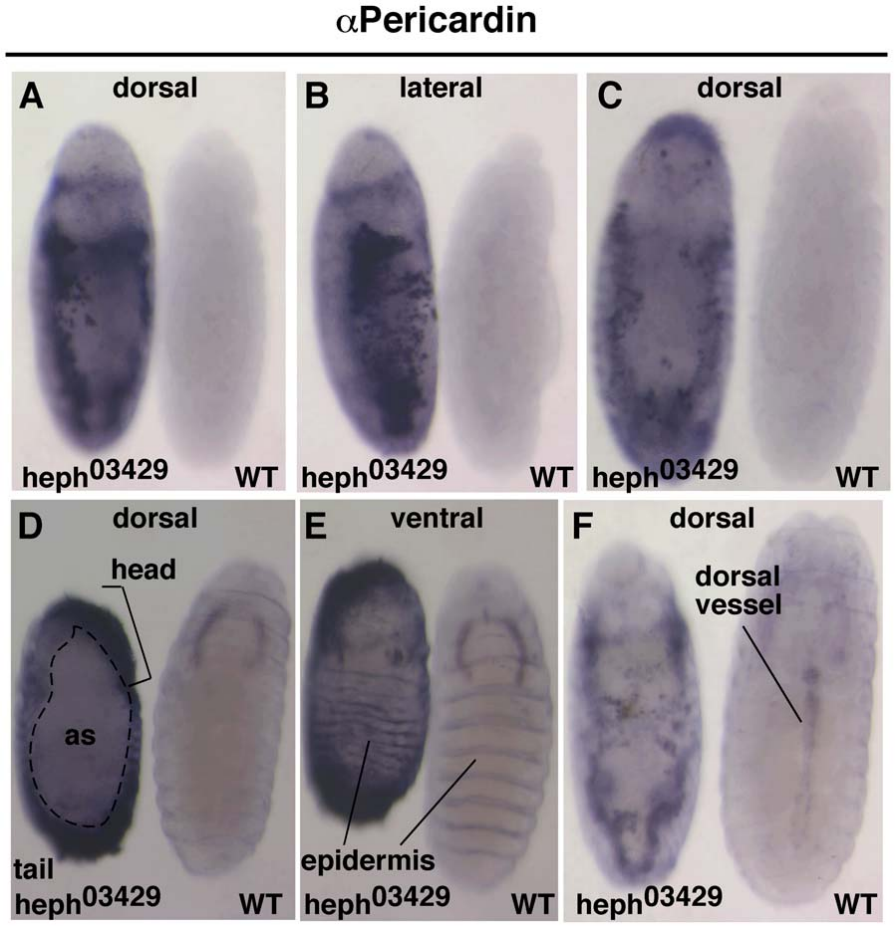
Pericardial cells are in excess and mislocalized in *heph^03429^* embryos indicating that cardiogenesis is disrupted in these embryos. **A**–**C**. The same *heph^03429^* and wild type embryos imaged from different perspectives. **D**. Pericardin was present in unusual places in *heph^03429^* embryos, such as the head and the tail. Note the large amnioserosal region (as) that persisted in *heph^03429^* embryos. **E**. The ventral epidermis was reasonably developed in *heph^03429^* embryos indicating that these embryos produced sufficient amounts of canonical Notch signaling in the ventral region. **F**. Pericardial cells continued to be in excess and cardiogenesis blocked in *heph^03429^* embryos at the time when embryogenesis and dorsal vessel formation was complete in wild type embryos (stage 17). All embryos shown were from the same experiment and were processed identically.

In *heph^03429^* embryos both the pericardial cell specification process (prior to dorsal closure) as well as migration of these cells to the dorsal mid-line (that is dependent on dorsal closure) were affected (**Fig. 4**). Figure **4A** and **4B** show two views of the same set of embryos; embryos in **4C** are from another set. Pericardin expression initiated prematurely, at stage 9, at which stage the wild type (WT) embryos have not even begun to express Pericardin (these embryos begin expression at stage 13). Pericardial cells were in high numbers and studies of numerous independently processed samples indicated that the pericardial cell specification process proceeded in a haphazard manner. For example, Pericardin was found in regions that do not normally express it, such as the head and the tail regions (see *heph^03429^* embryo in **4D**). Embryos in **4E** show the ventral region of the embryos in **4D** to point out that the ventral epidermis is developed, which is consistent with canonical Notch signaling activity in the region. Abnormal phenotypes in *heph^03429^* embryo persisted to the time when dorsal vessel is fully formed in the wild type embryo (embryos in **4F**). We observed similar defects in *heph^2^* embryos but they were milder or in a smaller fraction of embryos. These data indicate that the loss of *hephaestus* function affects not just the dorsal closure process but also additional processes that are dependent on the dorso-lateral regions of the embryo.

### 4. Notch expression is differently affected in ventral and dorso-lateral regions of mutant *hephaestus* embryos

**Figure 5:**
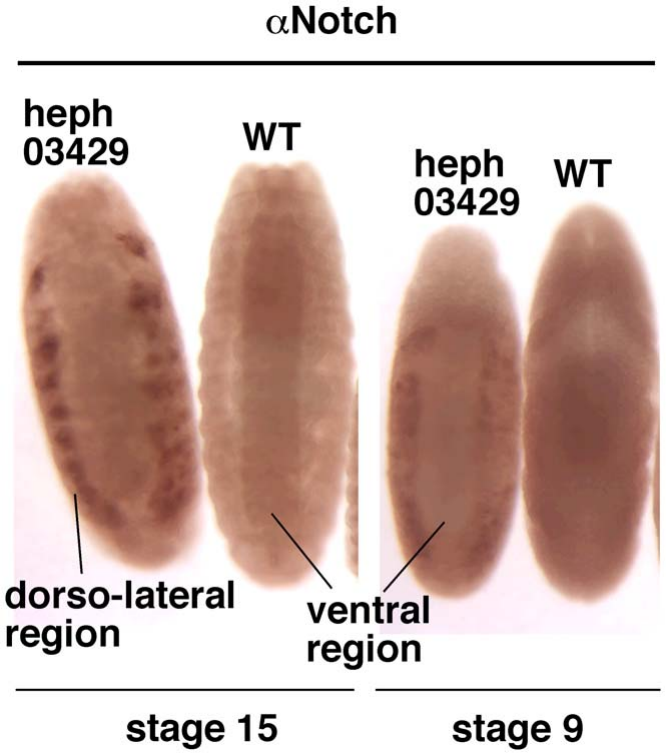
Notch protein level is different in different regions of *heph^03429^* embryos. **A**. Notch protein accumulated in the dorso-lateral region of stage 15 *heph^03429^* embryos but not in the ventral region. **B**. Accumulation of Notch protein in the dorso-lateral regions, and depletion in the ventral region, of *heph^03429^* embryos became apparent as early as Stage 9. All embryos shown were from the same experiment and were processed identically.

To determine whether loss of *hephaestus* function affected Notch protein levels, we studied *heph^03429^* embryos using an antibody made against the carboxyl terminus of the Notch protein [24]. We found a remarkable difference between the level of the Notch protein in the ventral and dorso-lateral regions of the same mutant embryo. Notch protein accumulated to a high level in the dorso-lateral regions of *heph^03429^* embryo but not in the ventral region. Notch accumulation was very pronounced in stage 15 embryos but could be detected even in stage 9 embryos when the effect of the zygotic loss of *hephaestus* function begins to manifest (**Fig. 5**). Notch protein expression in the ventral region of *heph^03429^* embryos was always lower than the level in wild type embryos of the same age. This feature is not clearly discernible in stage 15 embryos in Figure 5 due to the higher expression of Notch in the notochord just beneath the surface of wild type embryos (notochord is suppressed in *heph^03429^* embryos). However, it is clearly discernible in stage 9 embryos in which Notch protein expression is confined to the outer layer of cells. We observed the same pattern of Notch protein expression (higher level in the dorso-lateral regions and a lower level in the ventral region) even in *heph^2^* embryos but it was not as obvious and was observed in a smaller fraction of embryos.

### 5. Actin level is differently affected in ventral and dorso-lateral regions of mutant *hephaestus* embryos

**Figure 6:**
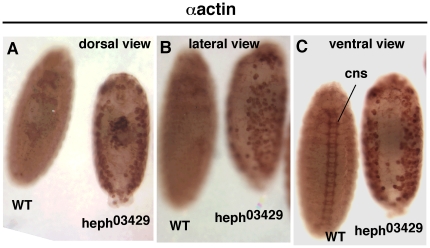
Actin level was high and disorganized in the dorso-lateral regions of *heph^03429^* embryos. **A, B, and C**: the same set of embryos shown from different perspectives. Absence of CNS labeling in the ventral region of the *heph^03429^* embryo is due to the absence of neuronal cells. Embryos are at stage 15. All embryos shown were from the same experiment and were processed identically.

**Figure 7:**
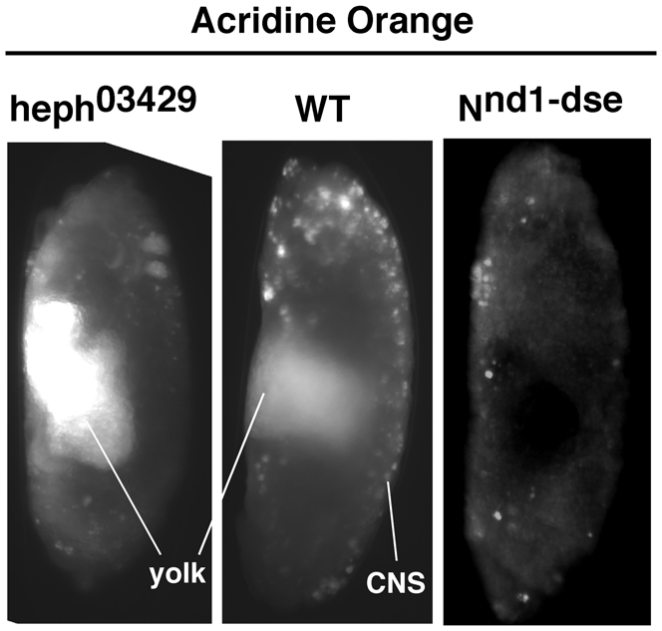
Apoptosis is not increased in *heph^03429^* and *N^nd1-dse^* mutant embryos. Normal apoptosis observed in the CNS of wild type embryo was not observed in either *heph^03429^* or *N^nd1-dse^* embryos as the CNS development in the latter embryos was suppressed. Embryos are at stage 14. The brightness of yolk is due to autofluorescence.

As the dorsal closure process is primarily dependent on actin-based processes [40] and Notch is known to be involved in actin-based processes [31], [36]–[38], we examined actin level in mutant *hephaestus* embryos. We found that actin accumulated to a high level and in a disorganized manner in the dorso-lateral regions of *heph^03429^* embryos (**Fig. 6**). In addition, we observed a depletion of actin in the ventral region of *heph^03429^* embryos compared to the level in wild type embryos (see Fig. 6B). We observed a similar pattern of actin accumulation in *heph^2^* embryos but it was less obvious and in a smaller fraction of embryos. To determine if actin accumulation in the dorso-lateral regions of *heph^03429^* embryos was due of increased apoptosis, we performed Acridine Orange labeling assay that reveals dying cells during Drosophila embryogenesis [44]. Results showed that *heph^03429^* embryos undergo apoptosis equal to or less than the wild type embryos of comparable stages (**Fig. 7**). It is well established that embryos homozygous for a null allele of the *wingless* gene experience increased cell death [52]. Therefore, we examined *wingless* null embryos and found that they do not accumulate actin in the dorso-lateral regions (**Fig. S4**). These results rule out cell death (apoptosis) as the cause of actin accumulation in mutant *hephaestus* embryos.

### 6. Notch and actin accumulation patterns overlap in mutant *heph^03429^* embryos

**Figure 8:**
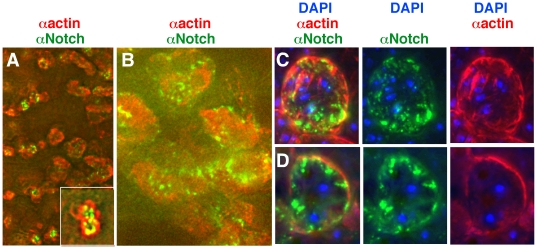
Notch and actin accumulated near the cell surfaces in the dorso-lateral regions of *heph^03429^* embryos. **A, B.** Low magnification images from DeltaVision restoration microscopy showing that Notch and actin expression overlap. **C, D.** Two sets of high magnification images from DeltaVision microscopy showing that Notch and actin accumulate near the surfaces of fused cells and not in the nucleus (marked by DAPI).

As Notch and actin protein accumulation patterns were comparable in the dorso-lateral regions of *heph^03429^* embryos, we examined whether there is overlap between Notch and actin accumulation. We performed high-resolution analysis using the DeltaVision restoration microscopy, which like confocal microscopy provides expression information in a single optical plane. We chose to focus on the dorso-lateral cells in late stage 15 *heph^03429^* embryos, as they show the highest level of Notch and actin accumulation. We used Alexa Fluor 488 conjugated and highly cross-adsorbed secondary antibody against the Notch antibody made in hamster, Alexa Fluor 647 conjugated and highly cross-adsorbed secondary antibody against actin antibody made in mouse, and DAPI for nuclear labeling. These fluorophores have no overlap in emission spectra. In all embryos we examined (n = 15), there was significant overlap in Notch and actin accumulation. Low magnification images of dorso-lateral regions of embryos are shown in **Figure 8A**–**B**. In many areas there was significant co-localization as shown by yellow color signals (inset in Fig. 8A) but in others there was none. Analysis at a higher magnification showed that Notch and actin accumulated at or near the cell surface, with little or no Notch in the nucleus (**Fig. 8C**–**D**). Interestingly, Notch and actin accumulation were across surfaces of many cells that appeared to have fused (see multiple DAPI signals in C and D). Cell fusion rather than defective cytokinesis appeared to be the explanation because the number of nuclei ranged from 2 to 11 nuclei (odd numbers are not expected with defects in cytokinesis). Furthermore, the sizes of nuclei were approximately the same within fusions and outside fusions indicating that multiple DAPI signals are not due to chromosomal fragmentation (**Fig. S5**). Thus, the loss of *hephaestus* function resulted in high levels of Notch, actin, and cell fusions.

### 7. N^intra^/NICD over-expression does not lead to abnormal Pericardin or actin levels

Accumulation of Notch could imply excess canonical Notch signaling. As shown in Figure S2, exogenous N^intra^/NICD over-expression mimics the classic gain-of-canonical-Notch-signaling phenotype: suppression of neurogenesis in the ventral region of *heph^03429^* embryos. Therefore, we examined if exogenous N^intra^/NICD expression also mimicked the high levels of Pericardin and actin in the dorso-lateral region of the embryo. Results of experiments examining pericardin level in UAS-N^intra^/NICD-daGal4 embryos are shown in **Figure S6**. We did not observe high levels of pericardin at any stage. In fact, pericardin level was suppressed. Comparable stages of wild type embryos showed normal Pericardin levels (embryos D–E). We obtained similar results with both daGal4 and hsGal4 drivers.

Results of our experiments examining actin level in UAS-N^intra^/NICD-hsGal4 embryos showed no effect at earlier stages (stages 11–12) but showed suppression of actin level at later stages (stages 13–14) (**Fig. S7A**). The latter result is the opposite of what we observed in mutant *heph^03429^* embryos. We also observed the loss of aminoserosa (AS) in N^intra^/NICD expressing embryos, which is the opposite of what we observed in *heph^03429^* embryos. We then examined embryos of the hyperactive Notch allele *l(1)N^B^*. This allele contains a mutation in the extracellular region that is a negative regulator of N^intra^/NICD production. As a consequence, N^intra^/NICD and canonical Notch signaling is produced at a high level in *l(1)N^B^/Y* embryos and these embryos die near the end of embryogenesis (stage 17) or in early larval stages ([28], [53], FlyBase). Results with *l(1)N^B^* embryos showed that actin level is not increased (**Fig. S7B**). We also did not observe increased levels of pericardin in *l(1)N^B^* embryos. In fact, we found that the dorsal vessel was missing or partially formed. These observations are in accord with our Gal4/UAS-N^intra^/NICD transgene data. Thus, it appears that while N^intra^/NICD and canonical Notch signaling are sufficient to explain the mutant phenotypes in the ventral region of *heph^03429^* embryos, they are insufficient to explain the mutant phenotypes in the dorso-lateral regions.

### 8. *N^nd1-dse^* embryos mimic the phenotypes of mutant *hephaestus* embryos

**Figure 9:**
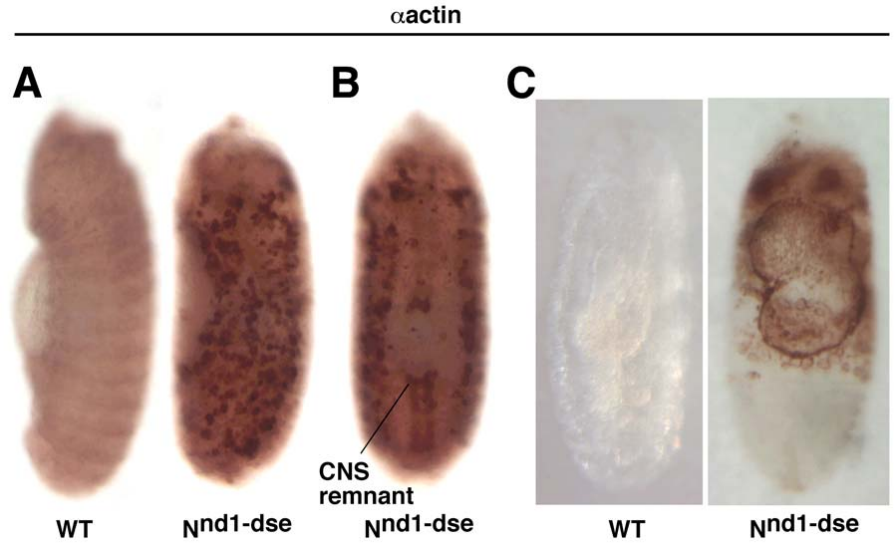
Embryos of the gain-of-Function Notch allele, *N^nd1-dse^*, manifest *heph^03429^*-like phenotypes. **A**. Actin accumulated in the dorso-lateral regions of *N^nd1-dse^* embryos. **B**. The CNS development is suppressed in the ventral region of *N^nd1-dse^* embryos. **C**. Dorsal closure was blocked in *N^nd1-dse^* embryos. Please compare *N^nd1-dse^* images in this figure with those of *heph^03429^* embryos in Figures 3 and 6. Embryos in A and B are at stage 14; the embryos in C are at stage 17 when embryogenesis ends in wild type embryos. All embryos were from the same experiment and were processed identically.

**Figure 10:**
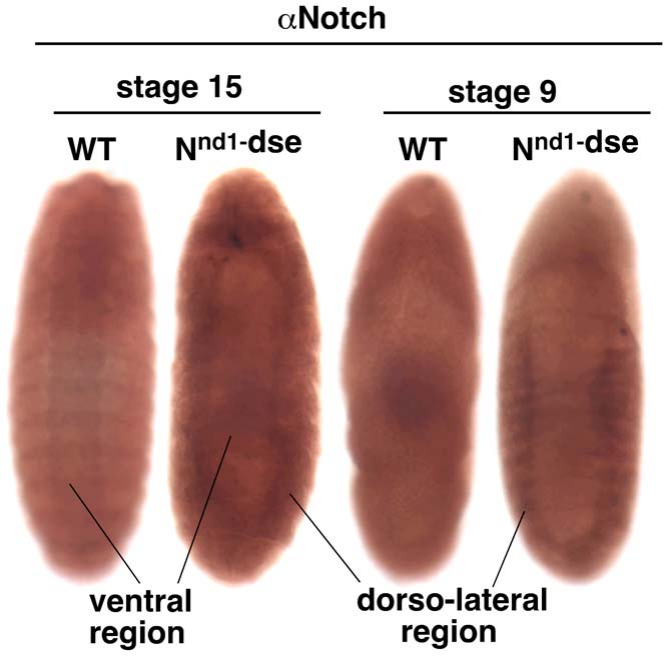
*N^nd1-dse^* embryos show Notch protein accumulation pattern that is similar to that observed in *heph^03429^* embryos. While the Notch protein accumulated in the dorso-lateral region, it was depleted in the ventral region. Depletion was more clearly discernible in stage 9 embryos that have not yet produced the notochord (that tends to obscure the depletion in images of embryos at later stages). Please compare *N^nd1-dse^* images in this figure with those of *heph^03429^* embryos in Figure 5. All embryos shown were from the same experiment and were processed identically.

The block in the dorsal closure process could be due to Notch accumulation in the dorso-lateral region that is disrupting actin metabolism. On the other hand, it could be unrelated to Notch accumulation. The latter was a distinct possibility since N^intra^/NICD over-expression failed to reproduce the dorsal closure or actin phenotypes. To distinguish between the two possibilities, we examined embryos of the temperature-sensitive *N^nd1-dse^* allele that produces constitutive and high levels of endogenous Notch activities at the restrictive temperature. The lesion in *N^nd1-dse^* is a mutation in the Down Stream Element (dse) that is critical for the negative regulation of Notch mRNA 3′ processing and protein production. At the restrictive temperature, more than two-thirds of the embryos die at various stages [18], [19]. If constitutive Notch activity is the cause of actin-related phenotypes in *heph^03429^* embryos, we expected to observe similar phenotypes in *N^nd1-dse^* embryos. We found that actin protein accumulated in the dorso-lateral regions of more than 50% of un-hatched *N^nd1-dse^* embryos that had developed past embryonic stage 14 (**Fig. 9A**). The same embryos also manifested suppression of CNS development in the ventral region and blocked dorsal closure process (**Fig. 9B, C**). Immuno-labeling with the Notch antibody showed that Notch protein also accumulated in the dorso-lateral regions of *N^nd1-dse^* embryos (**Fig. 10**). We checked for increased apoptosis in *N^nd1-dse^* embryos and found no evidence for it (please see Fig. 7). The similarities between *N^nd1-dse^* and *heph^03429^* phenotypes strongly suggested that the defects in the dorso-lateral regions of mutant *heph* embryos are caused by increased and/or constitutive Notch activity.

**Figure 11:**
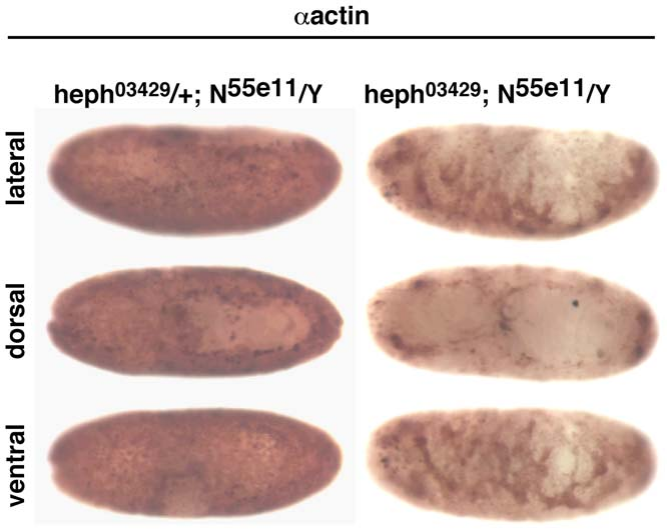
Reduction of Notch activity in *heph^03429^* embryos suppresses actin accumulation in the dorso-lateral regions. *N^55e11^* is a null allele of *Notch*. *heph^03429^*/+; *N^55e11^/Y* and *heph^03429^* ; *N^55e11^/Y* embryos were obtained from the same cross. +; *N^55e11^/Y* embryos are mostly composed of neuronal cells (with very few if any epidermal cells) and therefore show a higher level of actin compared to wild type embryos of similar stages. Suppression of actin level in the ventral region of *heph^03429^* ; *N^55e11^/Y* was due to rescue of epidermal tissue (a consequence of increased canonical Notch signaling). Actin accumulation was not observed in the dorso-lateral regions of these embryos.

Since Notch activity is required for producing epidermal cells, including those in the dorso-lateral regions, loss of Notch function can be expected to be epistatic to the loss of *hephaestus* function. However, since *hephaestus* is a negative regulator and the effect of the loss of Notch function in *Notch* zygotic null embryos manifests progressively from stages 9 to 13 (possibly due to variation in maternal contribution), we expected a fraction of *Notch*; *hephaestus* zygotic double mutant embryos to develop beyond stage 13. These embryos would enable us to determine if the actin accumulation in *hephaestus* mutant embryos is suppressed in the absence of *Notch*. Therefore, we generated double zygotic mutant embryos of *heph^03429^* and one of the Notch null alleles (*N^55e11^* and *N^264-47^*). About 70% of these embryos manifested the *Notch* null phenotype of excess neuronal cell types at the expense of epidermal cells. In other words, *Notch* is indeed epistatic to *hephaestus*, which is also consistent with data from studies in wing development indicating that *hephaestus* functions after Notch activation [17]. The remaining 30% showed varying degrees of suppression of *Notch* null phenotypes. A sample of embryos probed with the actin antibody is shown in **Figure 11**. Actin level was higher everywhere in *N^55e11^/Y* embryos than in comparable wild type embryos, possibly because they are composed of mostly neuronal cells. That high level of actin was drastically reduced when the embryos were also homozygous for the *heph^03429^*. Reduction in the ventral region was likely to be due to partial suppression of the neurogenic phenotype (possibly as a consequence of persistent canonical Notch signaling generated by maternal Notch protein). In the dorso-lateral regions of the same embryos, actin accumulation was not observed. We conclude from these observations that (1) actin accumulation in *heph^03429^* is dependent on Notch activity and (2) this Notch activity occurs later than the canonical Notch signaling activity in the ventral region. While these results are not conclusive (due to the complexity of *hephaestus* and *Notch* interactions), they are consistent with our hypothesis that *hephasestus* negatively regulates *Notch* that in turn positively regulates actin levels in the dorso-lateral regions of the Drosophila embryo.

### 9. Notch enrichment at the cell surface results in F-actin accumulation at the site

**Figure 12:**
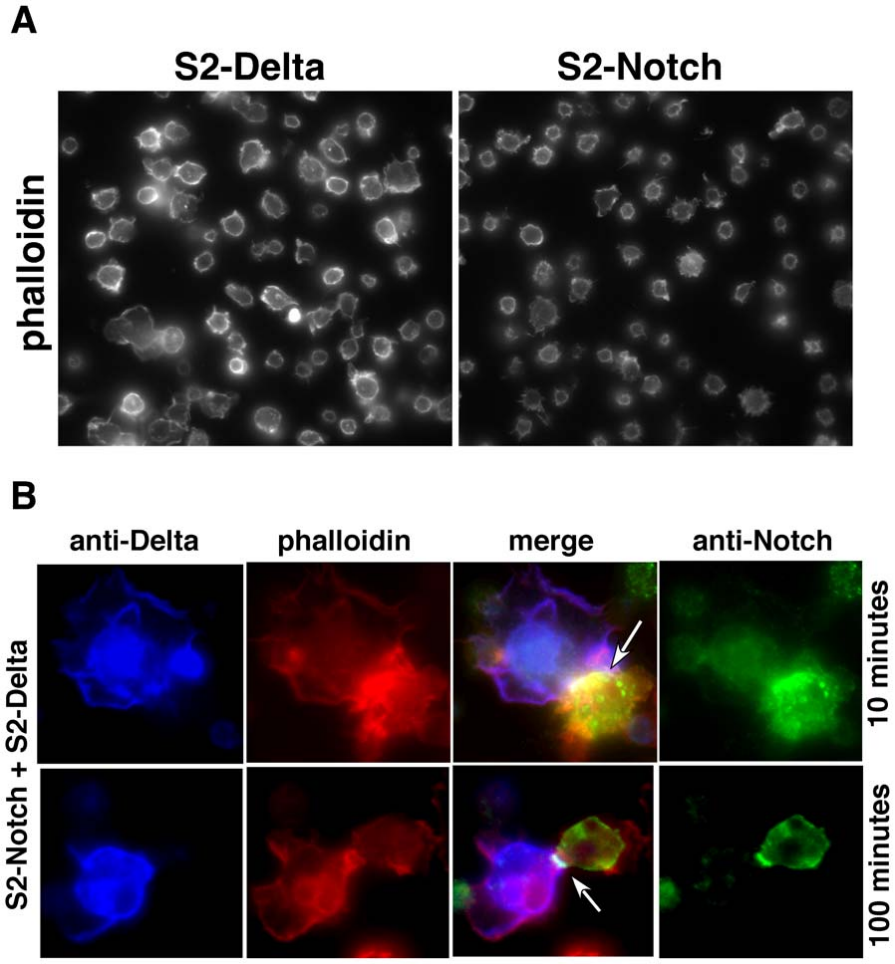
Enrichment of Notch receptor near the cell surface results in F-actin enrichment also near the cell surface. **A.** F-actin levels in S2 cells expressing Notch (S2-Notch cells) and S2 cells expressing ligand Delta (S2-Delta cells) that were not mixed together. **B.** F-actin levels in S2-Notch and S2-Delta cells brought together by Notch and Delta binding. Notch and Delta binding results in Notch receptor enrichment (clustering) at contact points between the two cell types (Delta does not show this response [27]). Notch enrichment diminishes over time, possibly due to production of N^intra^/NICD or reduction in Notch synthesis.

**Figure 13:**
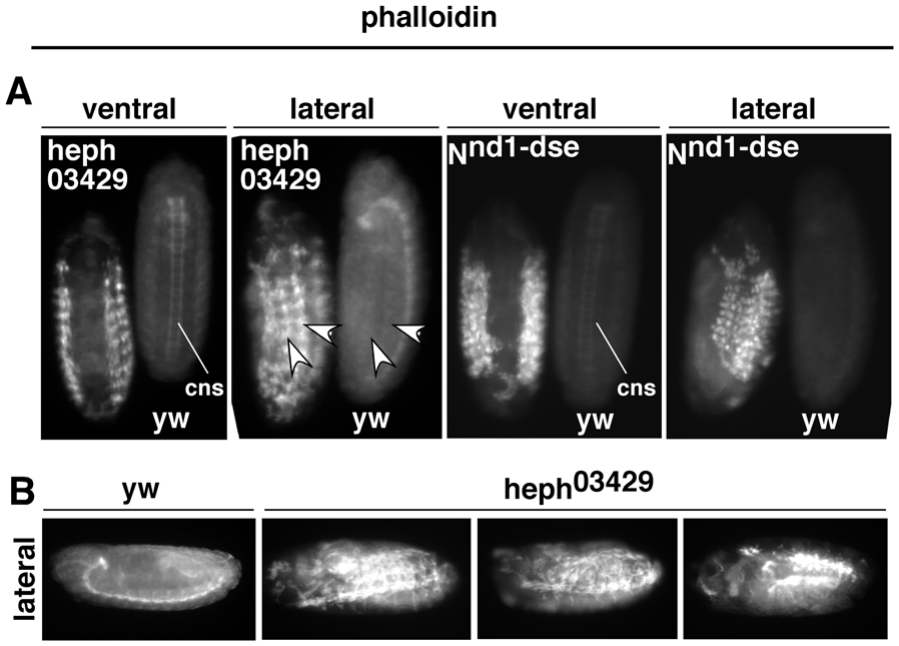
Phalloidin labeling indicates that actin that accumulates in the dorso-lateral regions of *heph^03429^* and *N^nd1-dse^* embryos is F-actin. **A.** Phalloidin labeling pattern in *heph^03429^* and *N^nd1-dse^* embryos resembled the actin antibody staining pattern (shown in Figures 6 and 9). Mutant and yw embryos were placed next to one another in a multi-well plate and imaged together. Arrowheads point to the ‘cable-like’ structures in *heph^03429^* and wild type embryos. **B.** F-actin accumulation in the dorso-lateral regions of *heph^03429^* embryos presented ‘cable-like’ patterns. Embryos were mounted individually and imaged under identical settings.

Since Notch and actin accumulated near the cell surface in *heph^03429^* and *N^nd1-dse^* embryos, we wondered if Notch accumulation at the cell surface results in actin accumulation. To find out we relied on the phenomenon of Notch receptor clustering, as it is the most natural way to enrich for Notch at the cell surface. When cultured Drosophila cells expressing Notch are treated with cells expressing a Notch ligand (e.g., Delta), the first and most rapid response is Notch receptor clustering at the sites of contact with ligand expressing cells. In the absence of the ligand expressing cells, Notch expression is distributed all around the cell membrane and in the cytoplasm. Delta does not cluster in response to Notch binding [27], [54]. We generated Notch clusters and examined actin levels using using Phalloidin that detects F-actin. Results showed clear enrichment of F-actin near Notch receptor clusters (**Fig. 12**). To determine if actin that accumulates in *heph^03429^* and *N^nd1-dse^* embryos is also F-actin, we probed these embryos with fluorescently labeled Phalloidin. These experiments confirmed that most of actin that accumulated in *heph^03429^* and *N^nd1-dse^* embryos is indeed F-actin (**Fig. 13A**). They also revealed a feature that was not apparent from actin antibody labeling: F-actin accumulates in longitudinal strings, as if the embryos are forming multiple longitudinal scaffolds (**Fig. 13B**). Such scaffolds are faintly visible even in the wild type embryos (see arrow heads in Fig. 13B) suggesting that normal scaffolds are enlarged in *heph^03429^* and *N^nd1-dse^* embryos. These results suggest that Notch accumulation at the cell surface has the potential to lead to F-actin accumulation in nearby locations, in the same pattern as in wild type embryos.

### 10. Persistent Notch-ligand interaction results in cell fusions *in vitro*

A glaring phenotype in the dorso-lateral regions of *heph^03429^* and *N^nd1-dse^* embryos is cell fusions. Although Notch is involved in processes that include cell fusions (for example, muscle development or cardiogenesis), whether it is directly involved in cell fusions as our data suggest is unknown. Therefore, we examined this issue in cultured cells that are simpler than embryos and have served as an excellent *in vitro* model system for the analysis of Notch and/or Delta activities for more than 20 years (for example [23], [24], [26]–[28], [54]–[57]).

**Figure 14:**
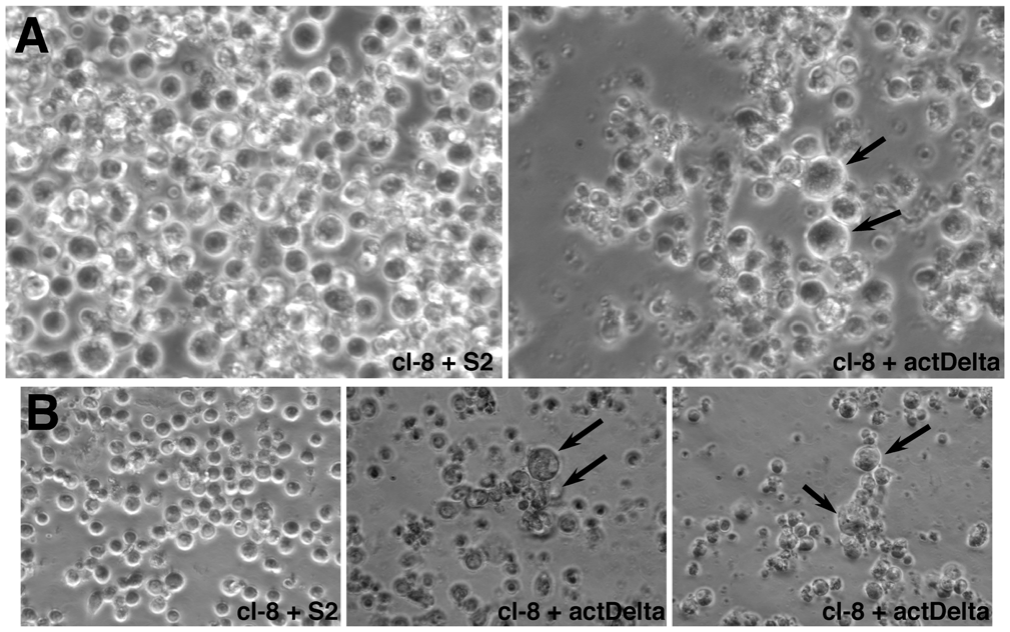
Large cells are produced in live clone-8 (cl-8) and Delta (actDelta) cell aggregates. **A**. Samples of cl-8 cells treated with S2 or actDelta cells at three million cells per milliliter density. **B**. Samples of cl-8 cells treated with S2 or actDelta cells at one million cells per milliliter density. Please note that Notch and Delta mediated cell aggregations are apparent only in cl-8+actDelta samples. Arrows point to large cells that formed within aggregates. Cell densities above one million per milliliter (required for the formation of cell aggregates) had no effect on the frequency of large cells.

We used clone 8 cells, that constitutively express Notch from the endogenous gene in order to retain as much of the natural regulations as possible, and S2-actDelta cells that constitutively express Delta from the acting5C promoter. Untransfected S2 cells (S2 cells) served as control cells. We treated cl-8 cells with either S2-actDelta cells or S2 cells for varying periods from 2 hours to 20 hours. After about 4 hours, we started to notice large cells. Samples from a high-cell-density experiment and a low-cell-density experiment are shown in **Figure 14**. These large cells became apparent only when cells were grown under natural conditions (in tissue culture treated plates or flasks) and were easily lost when the cell aggregates were washed or centrifuged.

**Figure 15:**
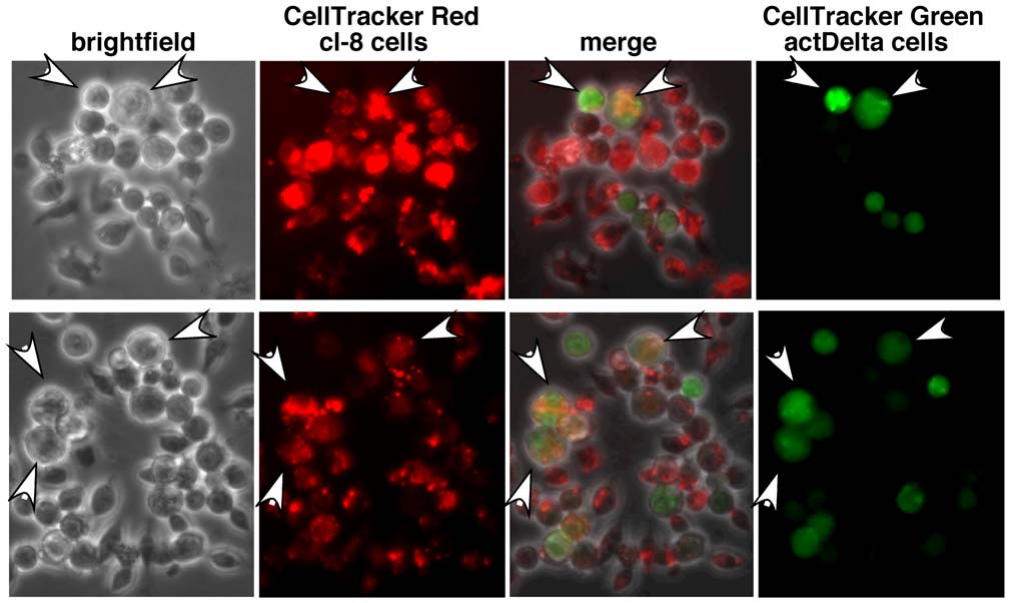
Large cells in live cl-8+actDelta cell aggregates are products of cell fusions. CellTracker Red labeled cl-8 cells and CellTracker Green labeled actDelta cells were used in the cell aggregation assay. These CellTracker dyes are confined to the treated cells or to their progeny upon division. The two rows represent samples from two independent assays. Arrowheads point to some fused cells. Note that other cells are either red or green and that there is no bleed-through between the red and the green channels.

To find out whether the large cells were products of cell fusion, we rendered clone 8 cells red and S2-actDelta cells or S2 cells green with cytoplasmic dyes that are confined to the treated cells for several cell generations. We mixed red clone 8 cells with either green S2-actDelta cells or green S2 cells, and processed them as described above but in the dark (to prevent fluorescence quenching). Double colored large cells indicating cell fusions became apparent in cl-8/S2-actDelta cell samples at about six hours and progressively increased over time. Eighty to ninety percent of cell aggregates (of 25–100 cells each) showed one or few fused cells (it is difficult to rule out fusion in the remaining aggregates as fused cells were easily dislodged). Two examples of cell fusion after 12 hours of incubation are shown in **Figure 15**. Mixtures of cl-8 cells and S2 cells that were processed identically do not aggregate and did not show evidence of cell fusion (**Fig. S8**). We have also not observed cell fusion in cells expressing N^intra^/NICD. These results suggest that persistent interaction between Notch and ligand (Delta) expressing cells (made possible only by binding between Notch and ligand {Delta} at the interface between cells) has the potential to lead to cell fusions and supports our observation of cell fusions in *heph^03429^* and *N^nd1-dse^* embryos. The low level of fusions observed *in vitro* could be due to lack of proper conditions or additional factors (fusogens) that promote cell fusions in embryos.

## Discussion

### 1. Novel aspects of canonical Notch signaling

Data presented in this report show that the loss of *hephaestus* (*dmPTB*) function affects the ventral and the dorso-lateral regions of Drosophila embryos very differently. In the ventral region, development of the CNS is suppressed and there was a discernible depletion in the level of the Notch protein. Suppression of the CNS development is consistent with the known role of *hephaestus* as a negative regulator of canonical Notch signaling during wing and bristle development in the larval and pupal stages [14], [15], [17]. Excess canonical Notch signaling is well known to suppress neurogenesis in embryos [58], [59]. Depletion of the Notch protein in the ventral region that could be explained using data from mammalian systems showing that N^intra^/NICD is turned over by a proteolysis process linked to the activity of the transcription factor Mastermind [29], [30]. Thus, excess canonical Notch signaling could result in Notch protein depletion if the rate of N^intra^/NICD production and degradation is higher than Notch synthesis. If that were the case, it would suggest that the mechanism responsible for down regulating Notch activity targets not N^intra^/NICD production or degradation but Notch synthesis. Combining the data from *heph* alleles and the *N^nd1-dse^* allele (presented here and in [18], [19]), it appears that most of Notch mRNA transcribed following Notch activation is targeted for degradation by a mechanism that requires the Notch 3′ UTR and the dse. It is possible that the Hephaestus protein is part of the RNP complex that regulates this mechanism. In its absence, Notch protein synthesis continues instead of being suppressed. There is growing evidence that ligand-independent canonical Notch signaling in involved in development [60], [61]. It would be interesting to know if this signaling is also affected by the Hephaestus-based down-regulation mechanism. Thus, understanding how exactly *hephaestus* negatively regulates canonical Notch activity might provide insights into an important aspect of Notch pathway regulation that was hitherto obscure: down-regulation after activation of Notch by a ligand. As many human diseases are linked to gain of canonical Notch signaling, a better understanding of *hephaestus* and *Notch* 3′ UTR and dse functions might lead to novel mechanistic insights into these diseases.

### 2. Revelations about a non-canonical Notch activity

The surprising finding in our study is the different response of the dorso-lateral regions of the embryo to the loss of *hephaestus* function or the loss of negative regulation of Notch mRNA 3′ processing (due to the *N^nd1-dse^* mutation). The simplest explanation is that Notch function is not required in these regions and de-repression of Notch protein synthesis results in the accumulation of Notch protein in these regions (as there is no signaling dependent depletion). This explanation, however, does not account for Pericardin accumulation, actin accumulation, or the block in dorsal closure. Pericardin level during cardiogenesis is well established to depend on Notch activity [50]. Our studies confirm that Pericardin is absent when Notch activity is eliminated (i.e., in *Notch* null embryos). Studies of others show that Notch activity is associated with higher actin level [31]. Thus, it is very likely that Notch function is required in the dorso-lateral region of the embryo and this function is in excess in mutant *heph* and *N^nd1-dse^* embryos.

As N^intra^/NICD expression does not lead to actin or Pericardin accumulation in the dorso-lateral region, the simplest explanation is that Notch function in the dorso-lateral region is not completely based on N^intra^/NICD activity in the nucleus. There is evidence for the existence of Notch activity independent of N^intra^/NICD. For example, a Notch function independent of Presenilin (the enzyme that is required for the release of N^intra^/NICD [20]–[22]) has been described in mammals [62]. A similar Notch activity might be functioning in the dorso-lateral regions of the Drosophila embryo. Our data suggest that this non-canonical Notch activity might be situated at the cell surface or in the cytoplasm and is involved in regulating actin levels and cell fusion. This inference is consistent with the finding of others that Notch activity other than the one based on N^intra^/NICD is associated with actin accumulation in wing discs [31].

The non-canonical Notch activity we appear to have discovered could be the predominant Notch activity in the dorso-lateral regions of the embryos but it cannot be the only Notch activity. It is well known that N^intra^/NICD and canonical Notch signaling is required for the peripheral nervous system (PNS) development from the dorso-lateral regions [20]–[22]. Our studies show that the PNS development is also suppressed in *heph^03429^* and *N^nd1-dse^* embryos, which raises the question of why we do not see depletion of Notch and actin proteins in the dorso-lateral regions as a consequence of constitutive N^intra^/NICD and canonical Notch signaling. There are two possible explanations. One, a minority of cells are involved in the PNS development. Two, the canonical Notch signaling activity might precede the non-canonical Notch signaling activity and the latter determines the ultimate phenotype. Regardless of which explanation is correct, it is remarkable that cells in the ventral and the dorso-lateral regions respond so differently to the loss of *hephaestus* function or to the loss of negative regulation of *Notch* activity due to the *N^nd1-dse^* mutation. At an earlier stage (stage 8 or 9), these cells were all the same, as they all adopt the default neuronal fate in *Notch* or *Delta* null embryos. At later stages, the ventral epidermal cells appear to diverge by blocking non-canonical Notch signaling altogether. It appears that this block is not an intrinsic lack of competence because actin and Notch enriched cells are occasionally observed in the ventral region (the *heph^03429^* embryo in Figure 6 was chosen to make this point; please see Fig. 6C). The block is specific to *hephaestus* or actin-related Notch activity as the ventral epidermal cells participate in other the actin-dependent processes, for example those involved in producing the denticle belts.

### 3. A clue to Notch function in the dorso-lateral regions of the embryo

The Notch pathway is long known to be involved in actin and adhesion processes in Drosophila. Interestingly, many processes that depend on *Notch* or *hephaestus* activity undergo cell fusion or block them (e.g., myoblast fusion [63] or spermatid individualization [64], please see [63] for a review of cell fusion). In this regard, Notch functions in myogenesis are quite instructive. During myogenesis, N^intra^/NICD and canonical Notch signaling is required to restrict the number of myoblasts. Not as well known is the fact that Notch activity is also required subsequently for myoblast fusion and differentiation [65]. A Notch activity at these stages is also reported to affect the differentiation of the neighboring epidermal cells and this activity is not based on N^intra^/NICD [66]. These reports have not been examined in depth so far because nothing is known about the non-canonical Notch signaling mechanism. The dorso-lateral regions of *heph^03429^* and *N^nd1-dse^* embryos represent an excellent model system for exploring non-canonical Notch mechanism with an unusual empirical power: all aspects of this mechanism in the dorso-lateral regions of the embryos can be compared with the canonical Notch signaling pathway mechanism in the ventral region *of the same embryo*.

**Figure 16:**
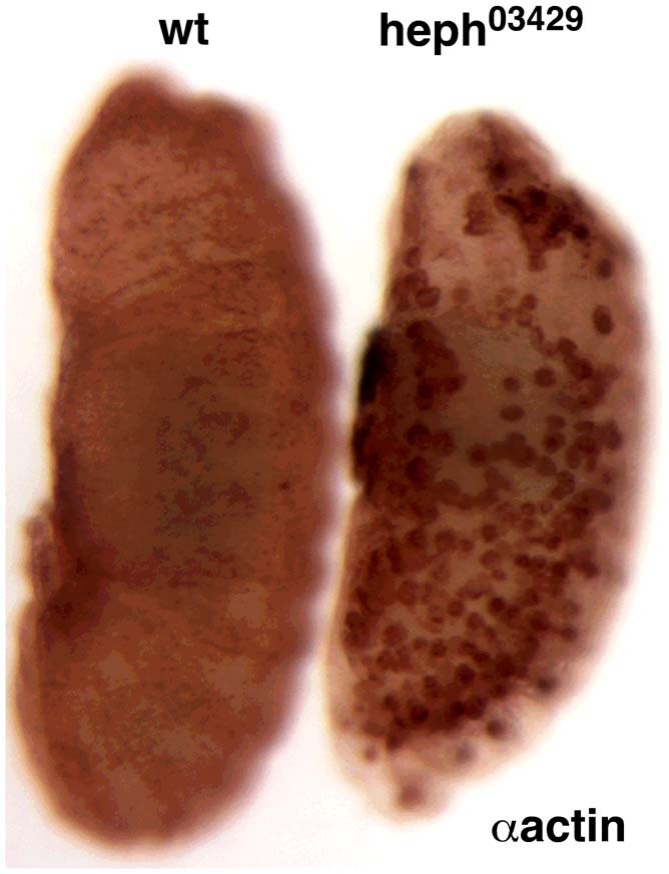
The pattern of actin accumulation in *heph^03429^* embryos resembles the pattern of normal actin levels in wild type embryos. The image of actin labeled wild type and *heph^03429^* embryos was contrast-enhanced to reveal the faint actin patterns in the wild type embryo.

We know precisely the process that is defective in the ventral region of *heph^03429^* and *N^nd1-dse^* embryos (neuronal cell fate specification) but we do not know anything about the process that is defective in the dorso-lateral regions. Our data contains two clues to the latter process. One clue is in **Figure 16**, which shows a contrast-enhanced image of wild type and *heph^03429^* embryos probed with the actin antibody. A close examination of this image reveals that the strong actin signals in the *heph^03429^* embryo are more or less amplified and expanded versions of the above-background actin signals in the wild type embryo. The second clue is in *heph^03429^* and *N^nd1-dse^* embryos probed with phalloidin. It appears that these embryos form enlarged versions of the cable-like actin structures that traverse almost the entire length of the dorso-lateral regions in the body of the wild type embryo (please see arrow heads in Fig. 13). It is quite possible that clusters of cells in the dorso-lateral regions undergo partial or full fusion to form actin scaffolds that maintain epithelia integrity during remodeling and migration. Hypertrophy of these actin scaffolds could be the defect in the dorso-lateral regions of *heph^03429^* and *N^nd1-dse^* embryos. At this juncture, we do not know the mechanism by which actin protein level is altered in these mutant embryos.

### 4. Clues to a higher level of developmental organization

*heph^03429^* and *N^nd1-dse^* embryos appear to reveal a new level of developmental organization: broad zones that are competent or refractory to non-canonical Notch signaling activity. We do not know what factors or mechanisms determine these zones. A diverse array of mechanisms is known to regulate Notch activity at the protein level, such as glycosylation, trafficking, and proteolytic processing [67]–[81]. It is possible that the ventral and the dorso-lateral regions differ in these mechanisms. Understanding the mechanism underlying the zonation of Notch activity in Drosophila embryos might also have practical implications since the Notch pathway is an important regulator of stem cell differentiation and cancer development. It might help us understand variations within and among stem cell or cancer populations. It is possible that certain populations are composed of cells with potential for only the canonical Notch signaling while others include cells with potential for both canonical and non-canonical Notch signaling. Such differences in potentials might explain why some stem cells just proliferate while others differentiate or why some cancer cells are begin while others are metastatic.

## Supporting Information

Figure S1 Manifestation of *heph^03429^* phenotypes (actin accumulation in the dorso-lateral regions) is delayed to stage 17 (end of embryogenesis) when the mother was heterozygous for the Green TM3 balancer with the *Ser^1^* mutant allele. If the mother was heterozygous for a null allele of *Notch*, *heph^03429^* embryos hatched into larvae (data not shown). *TM3actGFPSer^1^* homozygotes ceased development at about stage 6, were severely deformed, or died in the larval stages. Animals were arranged in a multi-well plate and imaged under brightlight and UV light with filter to detect GFP fluorescence.
(TIF)

Figure S2 Expression of Hunchback in Notch null embryos and in N^intra^/NICD-overexpressing embryos. **A**. Too many neural cells were produced in embryos lacking Notch function (*N^55e11^/Y*). **B**. Too few neural cells were produced in embryos expressing high levels of N^intra^/NICD and canonical Notch signaling. All embryos were from the same experiment and were processed identically.
(TIF)

Figure S3 Cardiogenesis (dorsal vessel formation) requires Notch function. Pericardial cells were not formed in *N^55e11^*/Y embryos that lack Notch function. All embryos were from the same experiment and were processed identically.
(TIF)

Figure S4 *wingless* null embryos that are known to experience increased apoptosis do not accumulate actin in the dorso-lateral regions. *wg^cx4^* is a null allele of *wingless*. Both embryos were from the same experiment and were processed identically.
(TIF)

Figure S5 The sizes of nuclei are similar inside and outside the regions of high actin accumulation in the dorso-lateral regions of *heph^03429^* embryos. This is the full DeltaVision image that was the source for Figure 8C. The similar nucleus sizes across the whole image indicate that multiple DAPI signals within rings of high actin expression are not due to chromosome fragmentation. The odd numbers of DAPI signals within such actin rings indicate fusion rather than defective cytokinesis.
(TIF)

Figure S6 N^intra^/NICD over-expression does not result in increased Pericardin level. Embryos from stage 9 to the end of embryogenesis were studied but only Stage 12 and 14 embryos are shown. Pericardin expression became apparent in wild type embryos only at stage 14. A stage 16 wild type embryo with fully formed dorsal vessel is also shown for comparison. All embryos were from the same experiment and were processed identically.
(TIF)

Figure S7 Excess canonical Notch signaling does not result in increased actin level. **A**. Excess canonical signaling due to N^intra^/NICD over-expression did not result in increased actin level. N^intra^/NICD over-expression did not affect actin level at early stages (stage 11) although other phenotypic consequences of its expression were apparent, such as the loss of aminioserosa (AS) or the block in germ-band retraction (resulting in the U-shaped phenotype commonly observed in embryos deficient for function of genes involved in germ-band retraction). However, N^intra^/NICD over-expression at later stages (stages 13–14) suppressed actin levels. **B**. Excess canonical Notch signaling due to expression of a hyper-active classical allele *l(1)N^B^* also did not result in increased actin level. Embryonic stages 13–14 are shown. All embryos were from the same experiment and were processed identically.
(TIF)

Figure S8 cl-8+S2 cell mixtures do not show evidence of cell fusion. CellTracker Red labeled cl-8 cells were treated with CellTracker green labeled S2 cells. Note that cells are either red or green.
(TIF)
