# Supplementary figures and images for: Loss of PTB or Negative Regulation of Notch mRNA Reveals Distinct Zones of Notch and Actin Protein Accumulation in Drosophila Embryo
Source: PLoS One. 2011 Jul 5;6(7):e21876. doi: 10.1371/journal.pone.0021876 (PMC3130057; doi:10.1371/journal.pone.0021876)

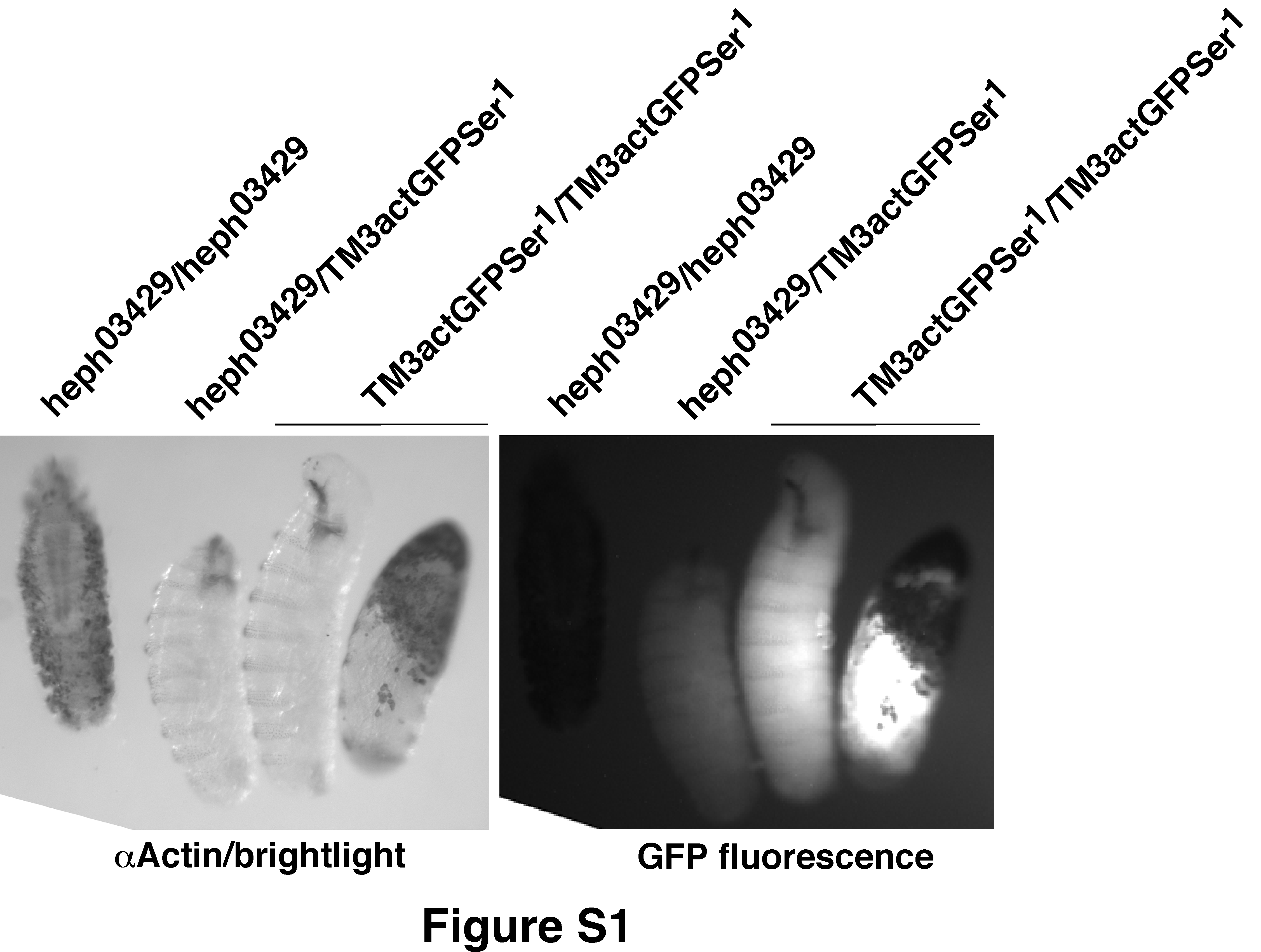

Supplement: Figure S1 — Manifestation of heph03429 phenotypes (actin accumulation in the dorso-lateral regions) is delayed to stage 17 (end of embryogenesis) when the mother was heterozygous for the Green TM3 balancer with the Ser1 mutant allele. If the mother was heterozygous for a null allele of Notch, heph03429 embryos hatched into larvae (data not shown). TM3actGFPSer1 homozygotes ceased development at about stage 6, were severely deformed, or died in the larval stages. Animals were arranged in a multi-well plate and imaged under brightlight and UV light with filter to detect GFP fluorescence. (TIF) [file pone.0021876.s001.tif]

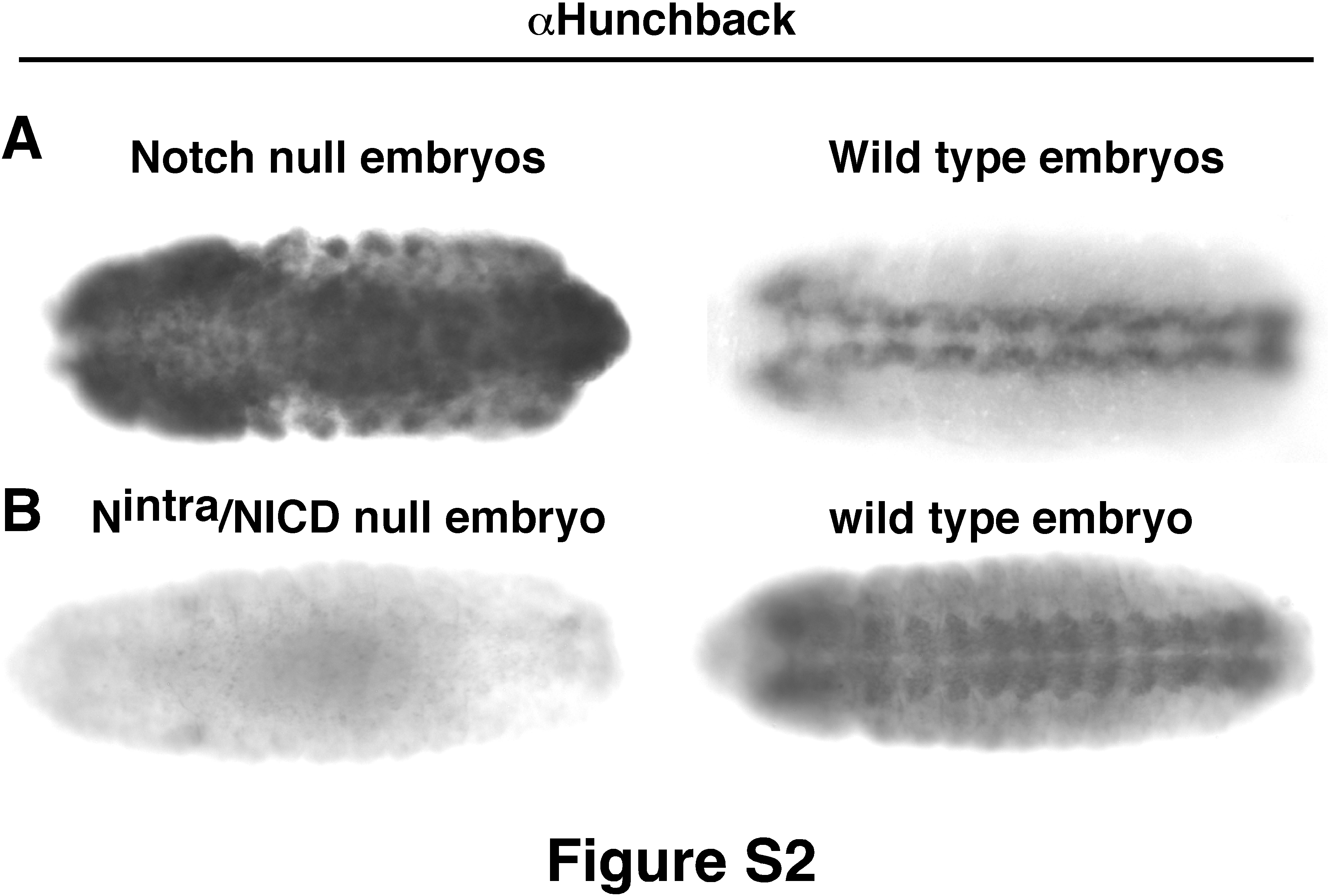

Supplement: Figure S2 — Expression of Hunchback in Notch null embryos and in Nintra/NICD-overexpressing embryos. A. Too many neural cells were produced in embryos lacking Notch function (N55e11/Y). B. Too few neural cells were produced in embryos expressing high levels of Nintra/NICD and canonical Notch signaling. All embryos were from the same experiment and were processed identically. (TIF) [file pone.0021876.s002.tif]

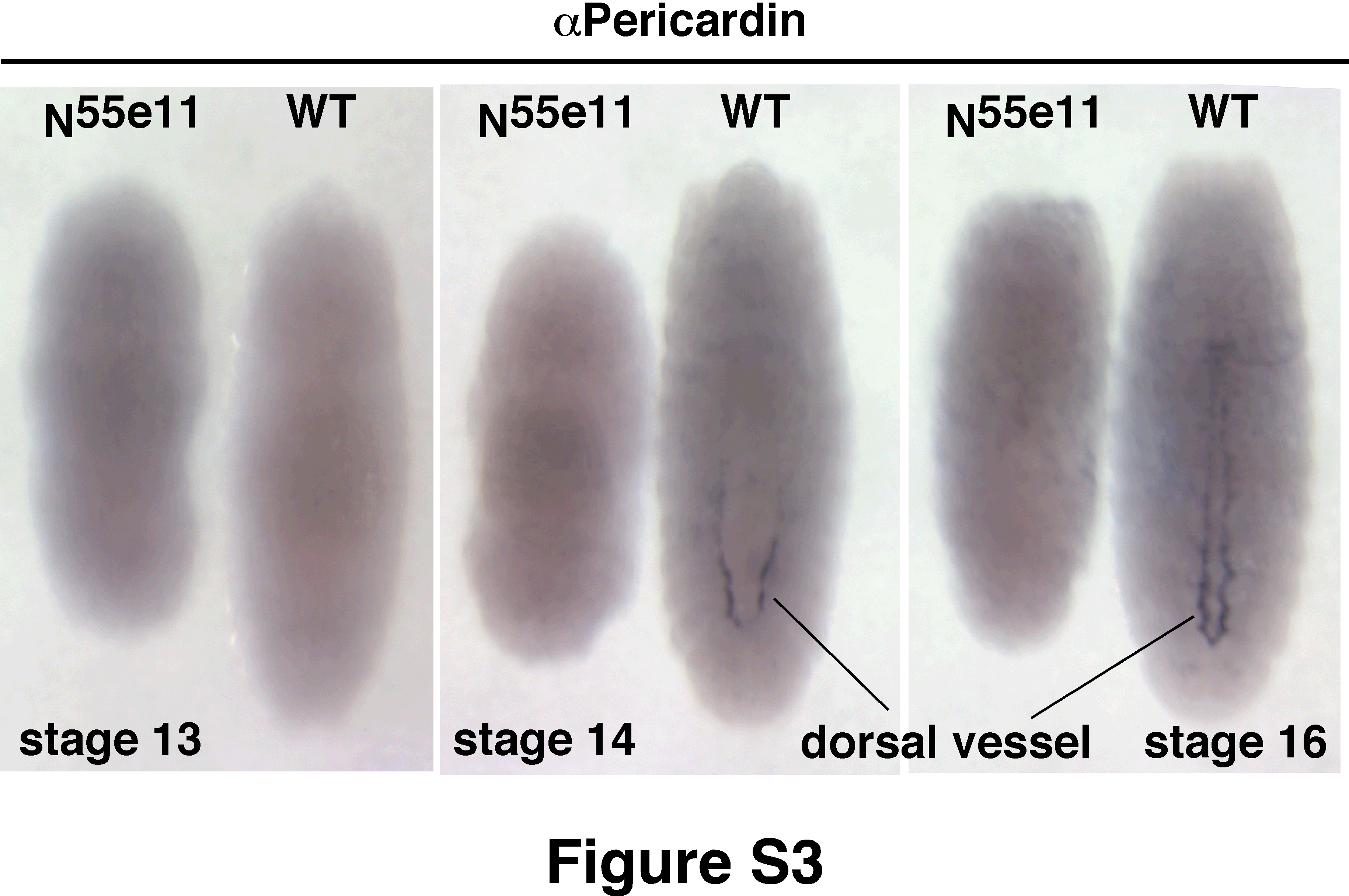

Supplement: Figure S3 — Cardiogenesis (dorsal vessel formation) requires Notch function. Pericardial cells were not formed in N55e11/Y embryos that lack Notch function. All embryos were from the same experiment and were processed identically. (TIF) [file pone.0021876.s003.tif]

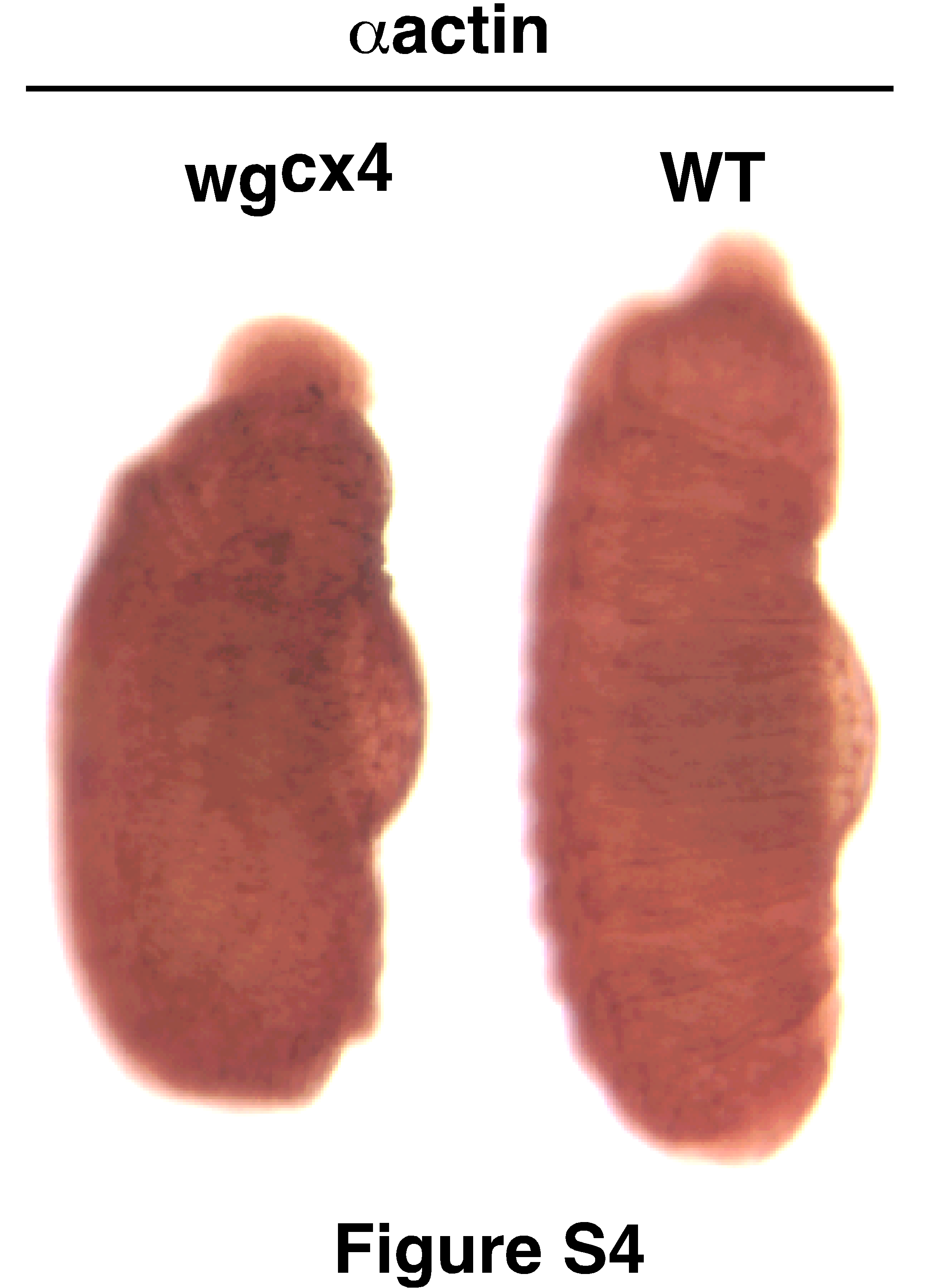

Supplement: Figure S4 — wingless null embryos that are known to experience increased apoptosis do not accumulate actin in the dorso-lateral regions. wgcx4 is a null allele of wingless. Both embryos were from the same experiment and were processed identically. (TIF) [file pone.0021876.s004.tif]

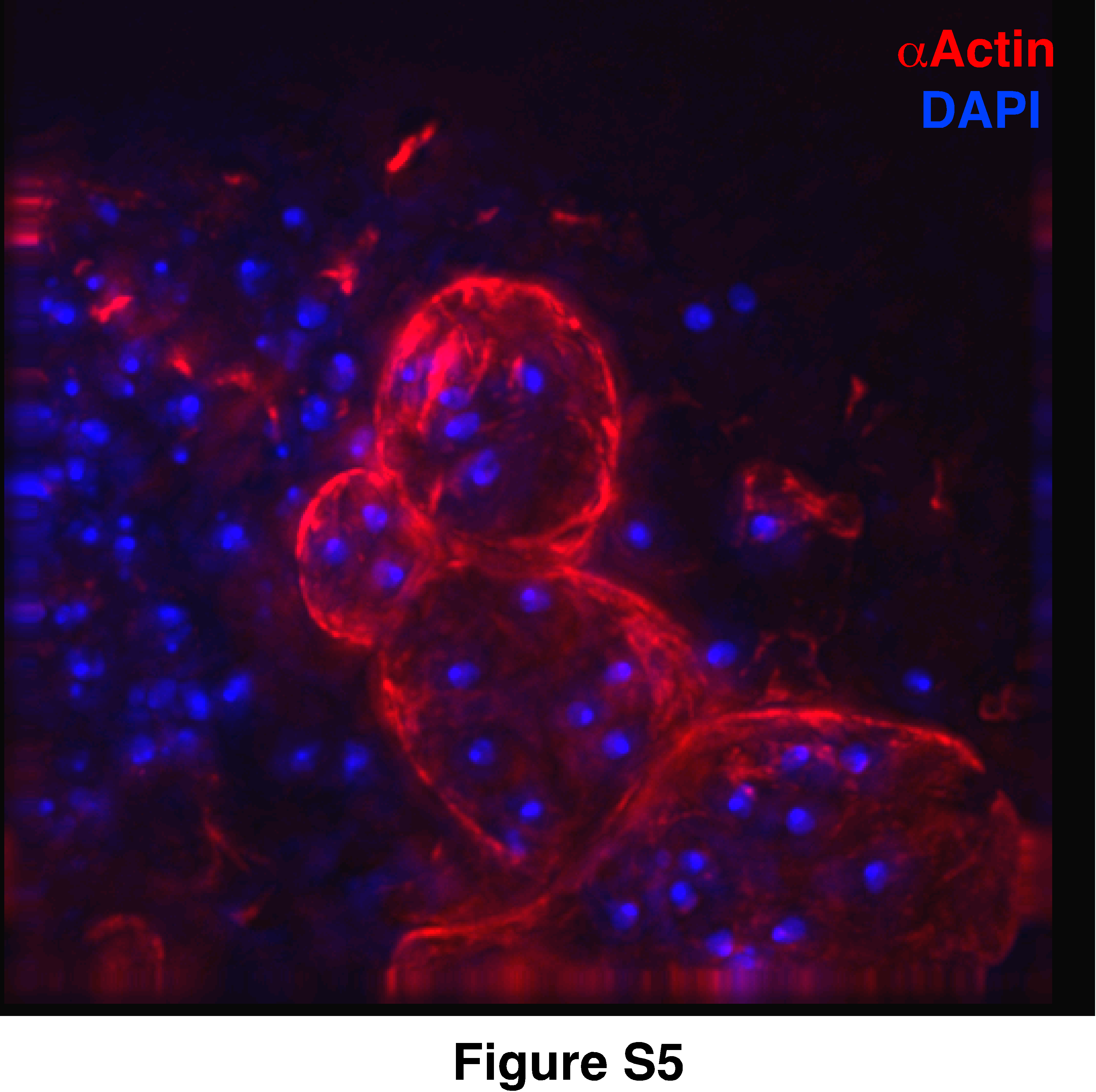

Supplement: Figure S5 — The sizes of nuclei are similar inside and outside the regions of high actin accumulation in the dorso-lateral regions of heph03429 embryos. This is the full DeltaVision image that was the source for Figure 8C. The similar nucleus sizes across the whole image indicate that multiple DAPI signals within rings of high actin expression are not due to chromosome fragmentation. The odd numbers of DAPI signals within such actin rings indicate fusion rather than defective cytokinesis. (TIF) [file pone.0021876.s005.tif]

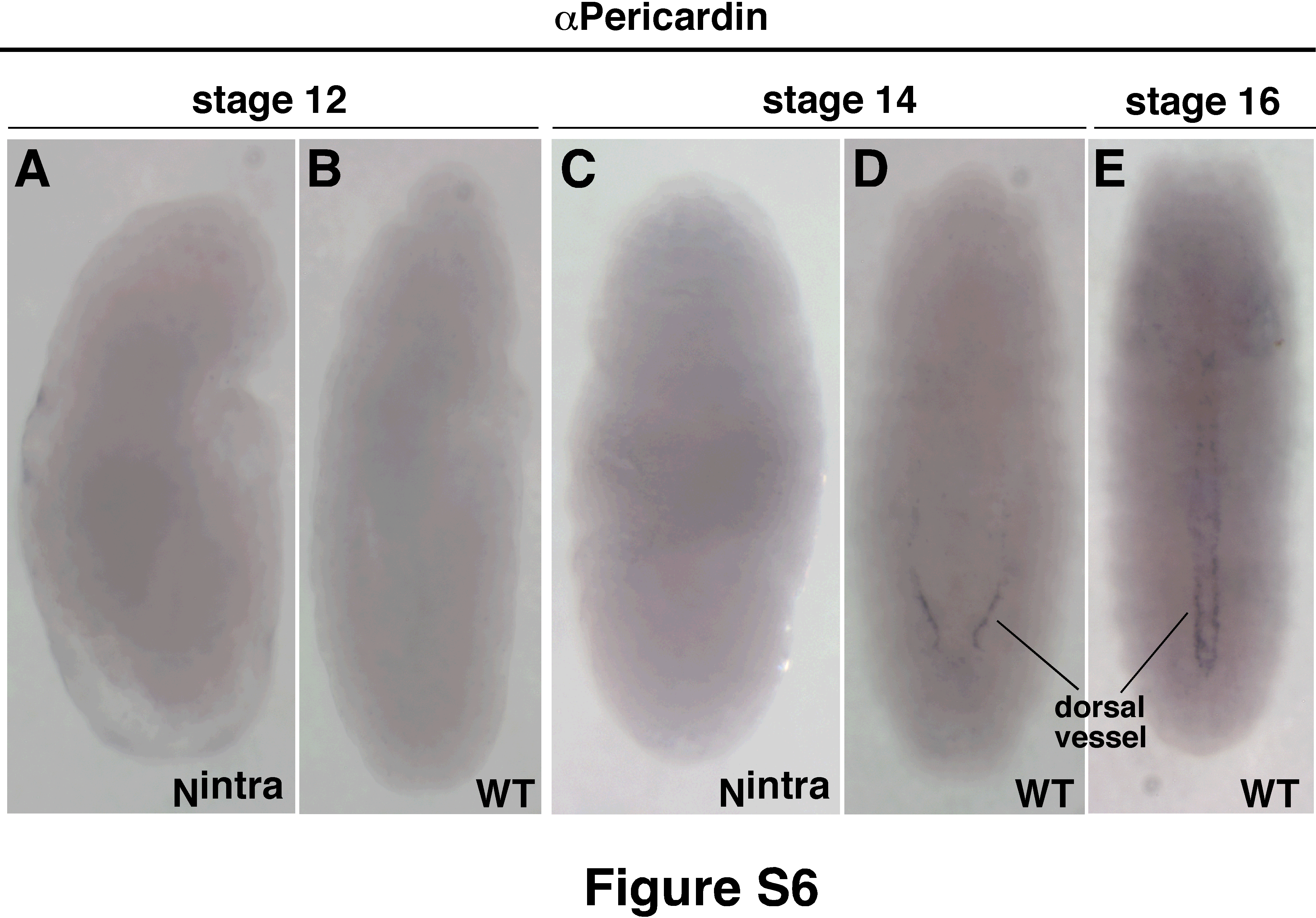

Supplement: Figure S6 — Nintra/NICD over-expression does not result in increased Pericardin level. Embryos from stage 9 to the end of embryogenesis were studied but only Stage 12 and 14 embryos are shown. Pericardin expression became apparent in wild type embryos only at stage 14. A stage 16 wild type embryo with fully formed dorsal vessel is also shown for comparison. All embryos were from the same experiment and were processed identically. (TIF) [file pone.0021876.s006.tif]

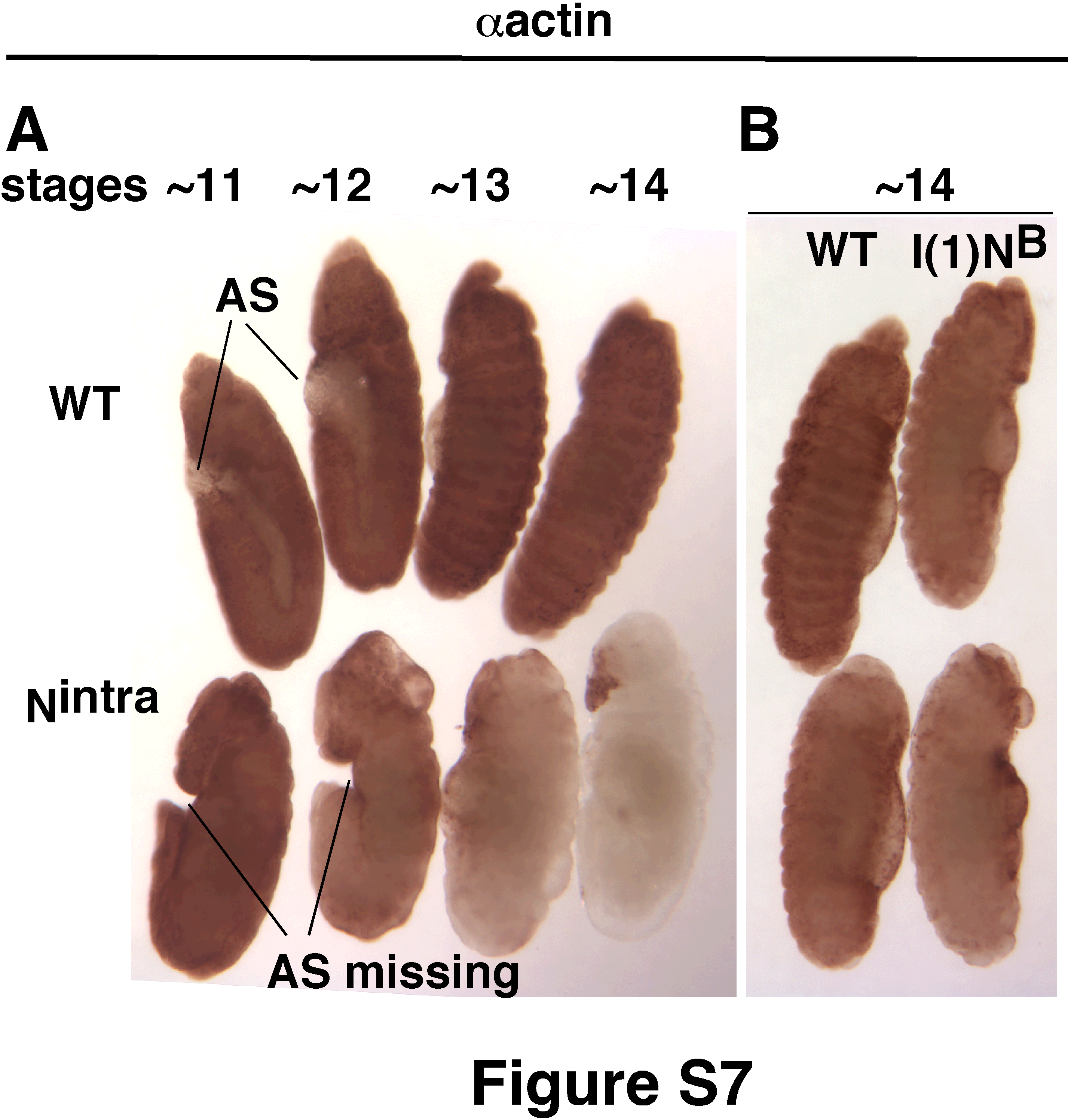

Supplement: Figure S7 — Excess canonical Notch signaling does not result in increased actin level. A. Excess canonical signaling due to Nintra/NICD over-expression did not result in increased actin level. Nintra/NICD over-expression did not affect actin level at early stages (stage 11) although other phenotypic consequences of its expression were apparent, such as the loss of aminioserosa (AS) or the block in germ-band retraction (resulting in the U-shaped phenotype commonly observed in embryos deficient for function of genes involved in germ-band retraction). However, Nintra/NICD over-expression at later stages (stages 13–14) suppressed actin levels. B. Excess canonical Notch signaling due to expression of a hyper-active classical allele l(1)NB also did not result in increased actin level. Embryonic stages 13–14 are shown. All embryos were from the same experiment and were processed identically. (TIF) [file pone.0021876.s007.tif]

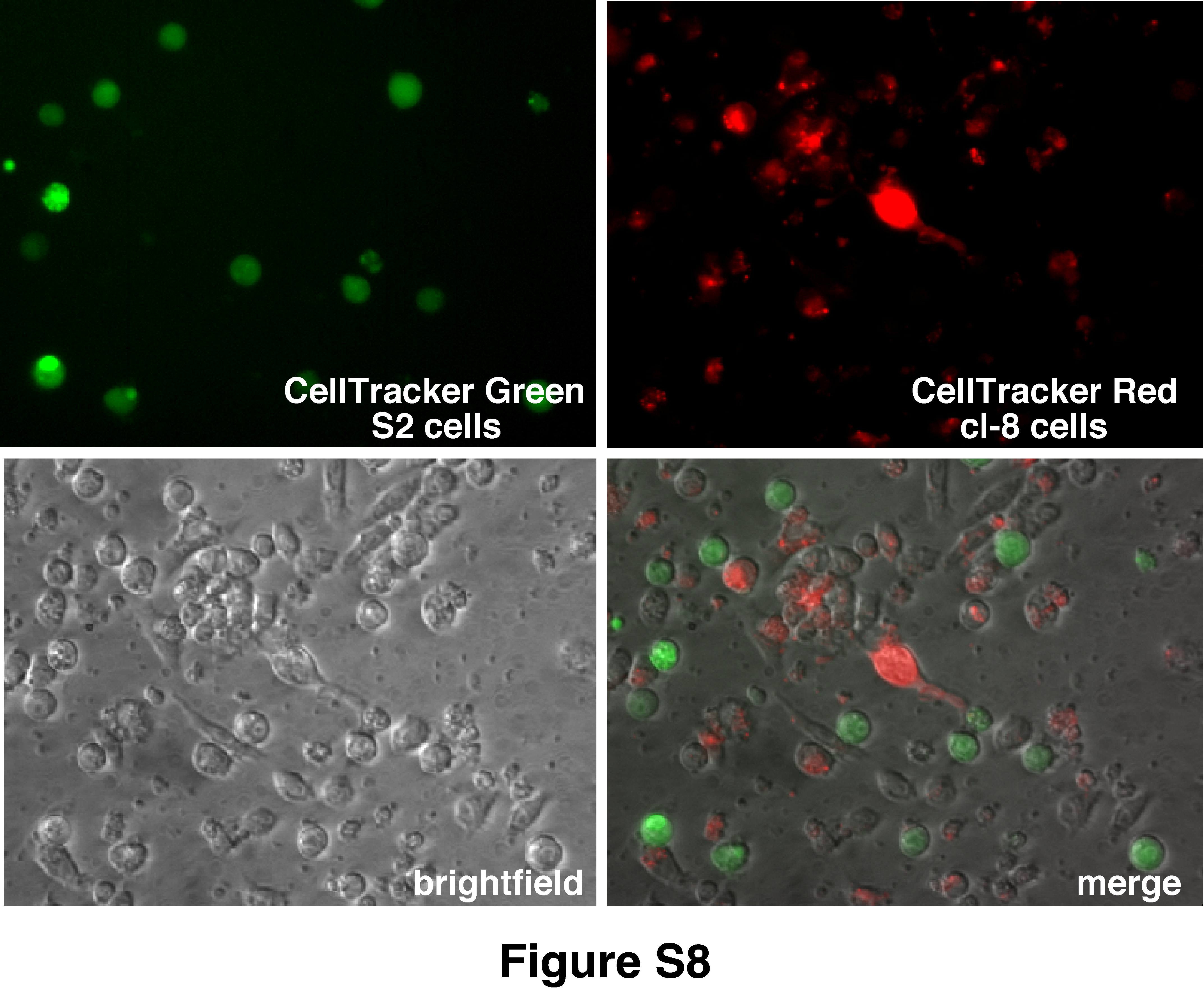

Supplement: Figure S8 — cl-8+S2 cell mixtures do not show evidence of cell fusion. CellTracker Red labeled cl-8 cells were treated with CellTracker green labeled S2 cells. Note that cells are either red or green. (TIF) [file pone.0021876.s008.tif]
